# Utility of 3-Acetyl-6-bromo-2*H*-chromen-2-one for the Synthesis of New Heterocycles as Potential Antiproliferative Agents

**DOI:** 10.3390/molecules201219803

**Published:** 2015-12-04

**Authors:** Sobhi M. Gomha, Yasser H. Zaki, Abdou O. Abdelhamid

**Affiliations:** 1 Department of Chemistry, Faculty of Science, Cairo University, Giza 12613, Egypt; s.m.gomha@hotmail.com; 2 Department of Chemistry, Faculty of Science, Beni-Suef University, Beni-Suef 62514, Egypt; Yzaki2002@yahoo.com

**Keywords:** pyrazolo[1,5-*a*]pyrimidine, tetrazolo[1,5-*a*]pyrimidine, 1,3,4-thiadiazoles, thiazoles, hydrazonoyl halides, hydroximoyl chlorides, antitumor activity

## Abstract

Coumarin derivatives containing pyrazolo[1,5-*a*]pyrimidine, tetrazolo[1,5-*a*]pyrimidine, imidazo[1,2-*a*]pyrimidine, pyrazolo[3,4-*d*]pyrimidine, 1,3,4-thiadiazoles and thiazoles were synthesized from 6-bromo-3-(3-(dimethylamino)acryloyl)-2*H*-chromen-2-one, methyl 2-(1-(6-bromo-2-oxo-2*H*-chromen-3-yl)ethylidene)hydrazine carbodithioate, 2-(1-(6-bromo-2-oxo-2*H*-chromen-3-yl)ethylidene)hydrazine carbothioamide and each of heterocyclic amine, hydrazonoyl chlorides and hydroximoyl chlorides. The structures of the newly synthesized compounds were elucidated on the basis of elemental analysis, spectral data, and alternative synthetic routes whenever possible. Moreover, selected newly synthesized products were evaluated for their antitumor activity against a liver carcinoma cancer cell line (HEPG2-1). The results revealed that pyrazolo[1,5-*a*]pyrimidine **7c**, thiazole **23g** and 1,3,4-thiadiazole **18a** (IC_50_ = 2.70 ± 0.28, 3.50 ± 0.23 and 4.90 ± 0.69 µM, respectively) have promising antitumor activity against liver carcinoma (HEPG2-1) while most of the tested compounds showed moderate activity.

## 1. Introduction

The synthesis of coumarins and their derivatives has attracted considerable attention from organic and medicinal chemists for many years as a large number of natural and synthetic products contain this heterocyclic nucleus. Coumarins possess diverse pharmacological and biological activities such as antitumor [1], analgesic and ulcerogenic [2], anti-inflammatory [3], anticoagulant [4], phototriggering [5], and fungicidal [6] properties, and can act as anticoagulants in the production of pesticides [7]. In particular, the antitumor activity of coumarin compounds has received considerable attention among researchers because of their cytotoxic activity against numerous types of cancers, including malignant melanoma, leukemia, renal cell carcinoma, prostate and breast cancer cell progression [8,9,10]. Also, certain platinum (II) complexes of aminocoumarins show very good *in vitro* cytotoxicity [11]. A variety of mechanisms have been proposed, such as interfering with estrogen synthesis, interfering with cell cycle progression or even acting as inhibitors of cytochrome P450 1 [12]. Despite numerous attempts to search for more effective antitumor agents, coumarins still a highly versatile class of compounds against cancer cell lines and are an important component among the molecules in drug discovery. The antitumor activities of coumarin were tested in several human tumor cell lines by Steffen *et al.* [13]. Both compounds inhibited cell proliferation of gastric carcinoma cell line (HSC-39), colon carcinoma cell line (Caco-2), hepatoma-derived cell line (Hep-G2) and lymphoblastic cell line (CCRF). Egan *et al.* [14] have synthesized, characterized and determined cytostatic and cytotoxic nature of 8-nitro-7-hydroxycoumarin using both human (including K-562 and HL-60) and animal cell lines grown *in vitro*. Warfarin reduces metastases from intestinal carcinomas to a great extent [15] and is also used as an adjunct to the surgical treatment of malignant tumors [16]. In addition, daphnetin inhibits tyrosine kinase, epidermal growth factor receptor, serine/threonine-specific protein kinase, and protein kinase C *in vitro* [17]. On the other hand, thiazole [18], 1,3,4-thiadiazole [19], azolo[1,5-*a*]pyrimidine [20], coumarin [21] derivatives displayed significant antitumor, cytotoxic, antiinflammatory, anticoagulant, antioxidant, antifungal, antitubercular, anticonvulsant, antimicrobial, antiviral, neuroprotective and diuretic activities. In continuation of our research program on the synthesis of novel heterocyclic compounds exhibiting antitumor activities [22,23,24,25,26], we attempted to design pyrazolo[1,5-*a*]pyrimidine, tetrazolo[1,5-*a*]-pyrimidine, imidazo[1,2-*a*]pyrimidine, pyrazolo[3,4-*d*]pyridazine, thiazoles, and thiadiazoles linked to position 3 of coumarin as a novel 3-heteroarylcoumarins, which have not been reported hitherto, to evaluate their *in vitro* antitumor activity against a liver carcinoma cell line (HEPG2-1).

## 2. Results and Discussion

### 2.1. Chemistry

**Scheme 1:**
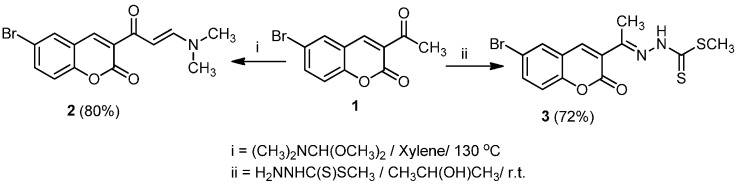
Synthesis of 6-bromo-3-(3-(dimethylamino)acryloyl)-2*H*-chromen-2-one (**2**) and methyl 2-(1-(6-bromo-2-oxo-2*H*-chromen-3-yl)ethylidene)hydrazine-1-carbodithioate (**3**).

Treatment of 3-acetyl-6-bromo-2*H*-chromen-2-one (**1**) [27] with each of *N*,*N*-dimethylformamide-dimethylacetal in boiling xylene and methyl hydrazinecarbodithioate in 2-propanol at room temperature yielded 6-bromo-3-(3-(dimethylamino)acryloyl)-2*H*-chromen-2-one (**2**) and methyl 2-(1-(6-bromo-2-oxo-2*H*-chromen-3-yl)ethylidene)-hydrazine-1-carbodithioate (**3**), respectively, in good yield (Scheme 1).

**Scheme 2:**
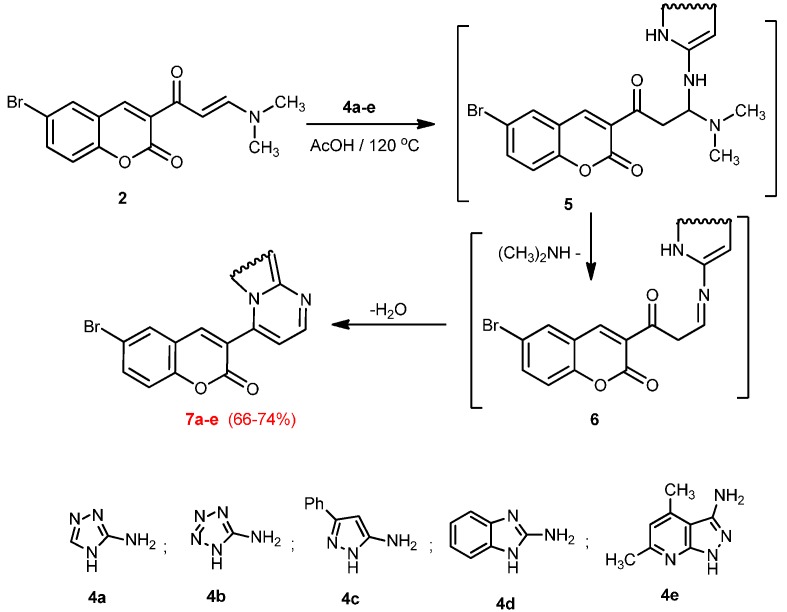
Synthesis of azolo[1,5-*a*]pyrimidine and imidazo[1,2-*a*]pyrimidine derivatives **7a**–**e**.

The structures of **2** and **3** were elucidated on the basis of elemental analysis, spectral data and chemical transformation. Thus, treatment of **2** with the appropriate 3-amino-1,2,4-triazole (**4a**), 5-amino-tetrazole (**4b**), 3-amino-5-phenylpyrazole (**4c**), 2-aminobenzimidazole (**4d**) or 4,6-dimethyl-1*H*-pyrazolo[3,4-*b*]pyridin-3-amine (**4e**) in acetic acid under reflux gave 3-([1,2,4]triazolo[1,5-*a*]pyrimidin-7-yl)-6-bromo-2*H*-chromen-2-one (**8a**), 6-bromo-3-(tetrazolo[1,5-*a*]pyrimidin-7-yl)-2*H*-chromen-2-one (**8b**), 6-bromo-3-(2-phenylpyrazolo[1,5-*a*]pyrimidin-7-yl)-2*H*-chromen-2-one (**8c**), 3-(benzo[4,5]imidazo[1,2-*a*]pyrimidin-4-yl)-6-bromo-2*H*-chromen-2-one (**8d**), and 6-bromo-3-(8,10-dimethylpyrido[2′,3′:3,4]pyrazolo[1,5-*a*]pyrimidin-4-yl)-2*H*-chromen-2-one (**8e**), respectively (Scheme 2). Structures **8a-e** were confirmed on basis of elemental analysis and spectral data.

**Scheme 3:**
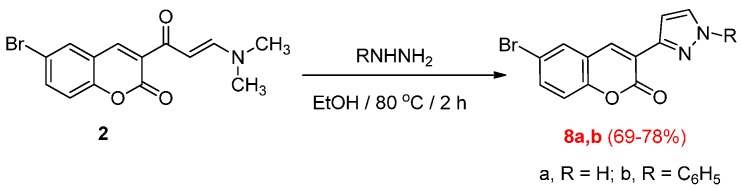
Synthesis of pyrazoles **8a** and **8b**.

Analogously, compound **2** was reacted with hydrazine hydrate and phenylhydrazine in ethanol to give 6-bromo-3-(1*H*-pyrazol-3-yl)-2*H*-chromen-2-one (**8a**) and 6-bromo-3-(1-phenyl-1*H*-pyrazol-3-yl)-2*H*-chromen-2-one (**8b**), respectively (Scheme 3).

**Scheme 4:**
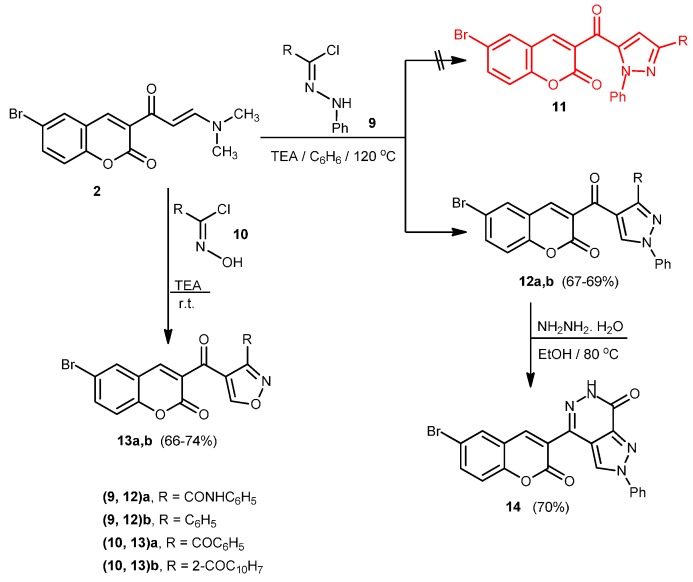
Synthesis of pyrazoles **12a**,**b**, isoxazoles **13a**,**b** and pyrazolo[3,4-*d*]pyridazone **14**.

Compound **2** was also reacted with 2-oxo-*N*-phenyl-2-(phenylamino)acetohydrazonoyl chloride (**9a**) in boiling benzene containing triethylamine to afford either 5-(6-bromo-2-oxo-2*H*-chromene-3-carbonyl)-*N*,1-diphenyl-1*H*-pyrazole-3-carboxamide (**11a**) or 4-(6-bromo-2-oxo-2*H*-chromene-3-carbonyl)-*N*,1-diphenyl-1*H*-pyrazole-3-carboxamide (**12a**) (Scheme 4). The structure of the product was elucidated on the basis of elemental analysis, spectral data and chemical transformation. The ^1^H-NMR spectrum showed at δ = 7.13–7.73 (m, 12H, Ar-H), 8.17 (s, 1H, Ar-H5), 8.40 (s, 1H, pyrazole-H), 8.54 (1H, (s, 1H, Ar-H4), 12.05 (s, D_2_O-exchangeable, 1H, NH). The product was reacted with hydrazine hydrate in boiling ethanol to give 4-(6-bromo-2-oxo-2*H*-chromen-3-yl)-2-phenyl-2,6-dihydro-7*H*-pyrazolo[3,4-*d*]pyridazin-7-one (**14**). Based on the above results the product was formulated as 4-(6-bromo-2-oxo-2*H*-chromene-3-carbonyl)-*N*,1-diphenyl-1*H*-pyrazole-3-carboxamide (**12a**) and structure **11a** was ruled out. Similarly, compound **2** was reacted with **9b**, **10a** and **10b** to afford 3-(3-benzoyl-1-phenyl-1*H*-pyrazole-4-carbonyl)-6-bromo-2*H*-chromen-2-one (**12b**), 3-(3-benzoylisoxazole-4-carbonyl)-6-bromo-2*H*-chromen-2-one (**13a**) and 3-(3-(2-naphthoyl)isoxazole-4-carbonyl)-6-bromo-2*H*-chromen-2-one (**13b**).

**Scheme 5:**
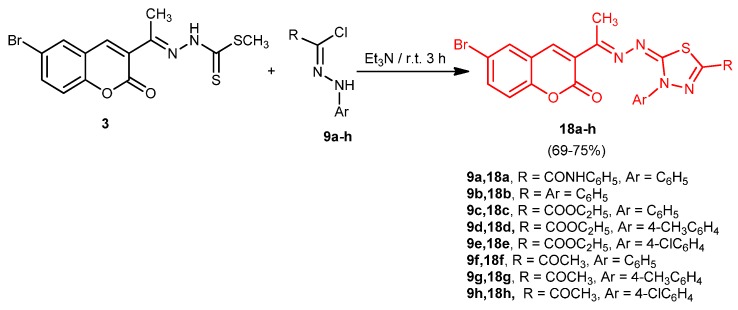
Synthesis of 1,3,4-thiadiazoles **18a**–**h**.

Next, *C*-ethoxycarbonyl-*N*-phenylhydrazonoyl chloride (**9c**) was reacted with methyl 2-(1-(2-oxo-2*H*-chromen-3-yl)ethylidene)hydrazine-1-carbodithioate (**3**) in ethanol containing triethylamine to afford ethyl 5-((1-(6-bromo-2-oxo-2*H*-chromen-3-yl)ethylidene)hydrazono)-4-phenyl-4,5-dihydro-1,3,4-thiadiazole-2-carboxylate (**18c**) (Scheme 5).

Structure **18c** was established by elemental analysis, spectral data, and alternative synthesis. Thus, ethyl 2-hydrazono-3-phenyl-1,3,4-thidiazoline-5-carboxylate [28] (**19**) reacted with **1** in ethanol to give a product identical in all aspects (m.p., mixed m.p. and spectra) with **18c**. Similarly, the appropriate **9a**,**b**,**d**–**h** was reacted with the appropriate **3** in ethanolic triethylamine to afford 2,3-dihydro-1,3,4-thiadiazoles **18a**,**b**,**d**–**h**, respectively.

**Scheme 6:**
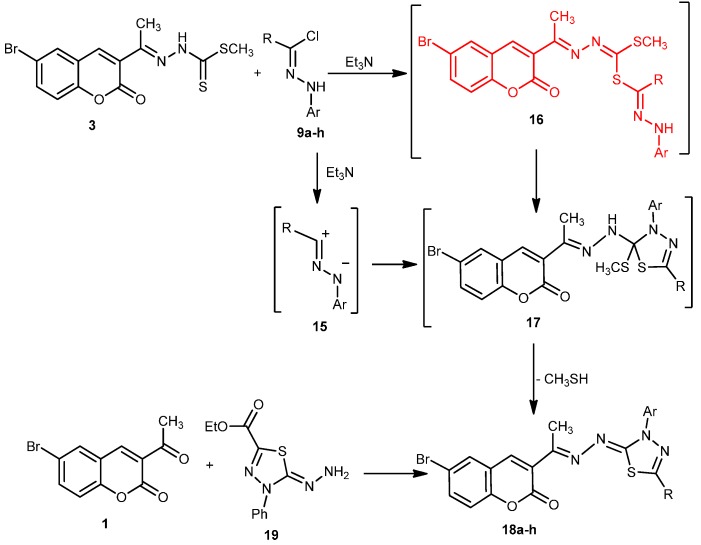
Mechanism of 1,3,4-thiadiazoles **18a**–**h**.

In the light of foregoing results, the mechanism outlined in Scheme 6 seems to be the most plausible pathway for the formation of **18** in the reaction of **3** with **9**. The reaction involves initial formation of thiohydrazonate **16**, which undergoes intramolecular cyclization as soon as it is formed to yield the intermediate **17** or undergoes 1,3-dipolar cycloaddition of nitrilimine **15** (generated *in situ* from **9** with triethylamine) to the C=S double bond of **3**. Compound **17** was converted to **18** by elimination of methyl mercaptan.

**Scheme 7:**
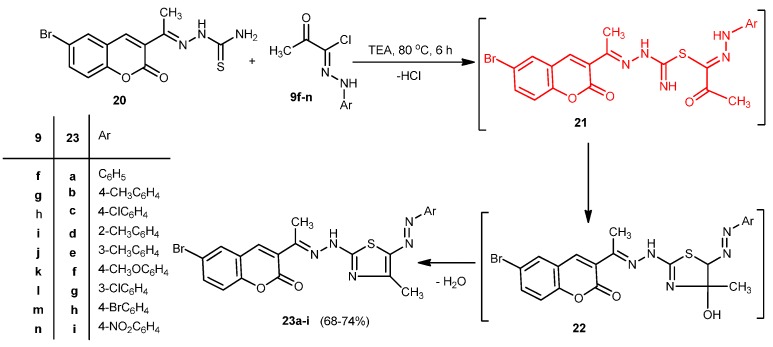
Synthesis of thiazoles **23a**–**i**.

Reaction of 2-(1-(6-bromo-2-oxo-2*H*-chromen-3-yl)ethylidene)hydrazine-1-carbothioamide [29] (**20**) with hydrazonoyl chloride **9f**–**n** in ethanol under reflux gave the corresponding thiazole derivatives **23a**–**i**, respectively, in good yield (Scheme 7). The structures **23a**–**i** were confirmed by elemental analysis and spectral data.

### 2.2. Antitumor Activity: Cytotoxicity against a Human Liver Carcinoma Cell Line (HEPG2-1)

The cytotoxic activity of 15 of the new compounds was determined against the liver carcinoma cell line HEPG2-1, using doxorubicin as a reference drug. Data generated were used to plot a dose-response curve of which the concentration (µM) of test compounds required to kill 50% of the cell population (IC_50_) was determined. Cytotoxic activity was expressed as the mean IC_50_ of three independent experiments (Table 1). The results revealed that the descending order of activity of the newly synthesized compounds was as follows: **7c** > **23g** > **18a** > **12a** > **23c** > **8a** > **7b** > **7e** > **18f** > **7a** > **7d** > **23d** > **12b** > **18c** > **13a**.

The pyrazolo[1,5-*a*]pyrimidine **7c**, thiazole **23g** and 1,3,4-thiadiazole **18a** (IC_50_ = 2.70, 3.50 and 4.90 µM, respectively) have promising antitumor activity against liver carcinoma (HEPG2-1) while the rest compounds have moderate activities (IC_50_ = 8.20 ± 1.54 − 17.4 ± 1.03 µM). On the other hand, isoxazole **13a** has poor inhibitory activity against (HEPG2-1) (IC_50_ = 15.3 ± 1.69 µM).

**Table 1:** Cytotoxic activities of tested compounds against liver carcinoma cell line (HEPG2-1).

Compound No.	IC_50_ (µM)	Compound No.	IC_50_ (µM)
**Doxorubicin**	1.40 ± 0.26	**12b**	17.1 ± 2.28
**7a**	14.2 ± 1.43	**13a**	15.3 ± 1.69
**7b**	10.0 ± 0.97	**18c**	17.4 ± 1.03
**7c**	**2.70 ± 0.28**	**18f**	13.0 ± 1.20
**7d**	14.6 ± 0.59	**18a**	**4.90 ± 0.69**
**7e**	12.8 ± 0.85	**23c**	9.10 ± 1.29
**8a**	9.80 ± 1.36	**23d**	15.5 ± 1.49
**12a**	8.20 ± 1.54	**23g**	**3.50 ± 0.23**

Examination of the SAR leads to the following conclusions:

- Among the fused pyrimidine derivatives, pyrazolo[1,5-*a*]pyrimidine **7c** is the most active one (IC_50_ = 2.70 ± 0.28 µM).
- Pyrazole derivative **12a** (substituted with CONHPh group at position 3) has *in vitro* inhibitory activity more than pyrazole derivative **12b** (substituted with Ph group at position 3)
- For 1,3,4-thiadiazole derivatives **18a**, **18c** and **18f**, compound **18a** (IC_50_ = 4.90 ± 0.69 µM) (with a chlorine atom as electron-withdrawing group on the aryl moiety) has promising antitumor activity while the other 1,3,4-thiadiazole derivatives **18c** and **18f** have moderate activities (IC_50_ = 17.4 ± 1.03 and 13.0 ± 1.20 µM, respectively).
- Among thiazole derivatives **23c**, **23d** and **23g**, compound **23g** (IC_50_ = 3.50 ± 0.23 µM) (with a chlorine atom as electron-withdrawing group on the aryl moiety) has promising antitumor activity, while the other thiazole derivatives **23c** and **23d** have moderate activities (IC_50_ = 9.10 ± 1.29 and 15.5 ± 1.49 µM, respectively).

## 3. Experimental Protocols

### 3.1. General Information

Melting points were measured on an Electrothermal IA 9000 series digital melting point apparatus (Bibby Sci. Lim. Stone, Staffordshire, UK). IR spectra were recorded in potassium bromide discs on Shimadzu FTIR 8101 PC infrared spectrophotometer (Shimadzu, Tokyo, Japan). The ^1^H- and ^13^C-NMR spectra were recorded on a Varian 6 Mercury VX-300 NMR spectrometer (Varian, Inc., Karlsruhe, Germany). ^1^H-NMR (300 MHz) and ^13^C-NMR spectra were recorded on a BRUKER spectrometer (Bruker BioSpin GmbH, Rheinstetten, Germany) in DMSO-*d*_6_ and chemical shifts are expressed in ppm units using TMS as an internal reference. Mass spectra were recorded on a Shimadzu (Tokyo, Japan) GCMS-QP1000 EX mass spectrometer at 70 eV. Elemental analyses were measured by using a German made Elementar Vario EL III CHNS analyzer (GmbH & Co. KG, Hanau, Germany). Antitumor activity was evaluated at the Regional Center for Mycology and Biotechnology at Al-Azhar University, Cairo, Egypt. Hydrazonoyl halides **9a**–**n** [30,31,32,33,34] were prepared as reported in the literature.

### 3.2. Chemistry

#### 3.2.1. Synthesis of 6-Bromo-3-(3-(dimethylamino)acryloyl)-2*H*-chromen-2-one (**2**)

A mixture of the 3-acetyl-6-bromo-2*H*-chromen-2-one (**1**, 2.67 g, 10 mmol) and *N*,*N*-dimethylformamide dimethyl acetal (DMF-DMA, 1.19 g, 10 mmol) in dry xylene (30 mL) was refluxed for 3 h, then allowed to cool. The solid product was collected by filtration, washed with petroleum ether (60/80 °C), dried and crystallized from ethanol to afford compound **3** as a pale yellow solid in 80% yield; mp 203–205 °C; IR (KBr) ν (cm^−1^): 3111, 3073, 3019 (=C–H), 2902 (–C–H), 1727, 1639 (2C=O), 1605 (C=N); ^1^H-NMR (DMSO-*d*_6_) δ: 2.86 (s, 3H, CH_3_), 3.14 (s, 3H, CH_3_), 5.83 (1H, d, *J* = 12.4 Hz, CH=CHCO), 7.37 (d, 1H, *J* = 9 Hz, Ar-H8), 7.72 (d, 1H, *J* = 9 Hz, Ar-H7), 7.79 (s, 1H, Ar-H5), 8.11 (d, 1H, *J* = 12.4 Hz, CH=CHCO), 8.38 (s, 1H, Ar-H4); MS *m*/*z* (%): 321, 323 (M^+^, M + 2, 37). Anal. Calcd for C_14_H_12_BrNO_3_ (322.15): C, 52.20; H, 3.75; N, 4.35. Found: C, 52.12; H, 3.68; N, 4.31.

#### 3.2.2. Synthesis of Methyl 2-(1-(6-Bromo-2-oxo-2*H*-chromen-3-yl)ethylidene)hydrazine carbodithioate (**3**)

To a solution of 3-acetyl-6-bromo-2*H*-chromen-2-one (**1**, 2.67 g, 10 mmol) in 2-propanol (20 mL), methyl hydrazinecarbodithioate **2** (1.22 g, 10 mmol) was added. The mixture was stirred at room temperature for 2 h. The solid product was filtered off, recrystallized from ethanol to afford **3** as a yellow solid in 72% yield; mp 187–189 °C; IR (KBr) ν (cm^−1^): 3337 (NH), 3092, 3066, 3041 (=C–H), 2980 (–C–H), 1735 (C=O), 1605 (C=N); ^1^H-NMR (DMSO-*d*_6_) δ: 2.49 (s, 3H, CH_3_), 2.58 (s, 3H, CH_3_), 3.30 (1H, s, NH), 7.40 (d, 1H, *J* = 9 Hz, Ar-H8), 7.85 (d, 1H, *J* = 9 Hz, Ar-H7), 8.18 (s, 1H, Ar-H5), 8.57 (s, 1H, Ar-H4); MS *m*/*z* (%): 370, 372 (M^+^, M + 2, 64). Anal. Calcd for C_13_H_11_BrN_2_O_2_S_2_ (371.27): C, 42.06; H, 2.99; N, 7.55; Found C, 42.01; H, 2.86; N, 7.49.

#### 3.2.3. Reactions of Enaminone **2** with Heterocyclic Amines **4a**–**e**

A mixture of enaminone **2** (0.322 g, 1 mmol) and 3-amino-1,3,4-triazole (**4a**), 5-aminotetrazole (**4b**), 3-amino-5-phenylpyrazole (**4c**), 2-aminobenzimidazole (**4d**), and 4,6-dimethyl-1*H*-pyrazolo[3,4-*b*]-pyridin-3-amine (**4e**) (1 mmol) in acetic acid (15 mL) was refluxed for 6–8 h. The reaction mixture was cooled and diluted with MeOH and the solid product was collected by filtration and recrystallized from dioxane to give **7a**–**e**, respectively.

*3-([1,2,4]Triazolo[1,5-a]pyrimidin-7-yl)-6-bromo-2H-chromen-2-one (***7a**): Brown solid, 69% yield, mp 275–278 °C; IR (KBr) ν (cm^−1^): 3097, 3055 (=C–H), 2960 (–C–H), 1726 (C=O), 1597 (C=N); ^1^H-NMR (DMSO-*d*_6_) δ: 7.37 (d, 1H, *J* = 9 Hz, Ar-H8), 7.72 (d, 1H, *J* = 9 Hz, Ar-H7), 7.79 (s, 1H, Ar-H5), 8.04 (1H, d, *J* = 4.5 *Hz*, pyrimidine-H), 8.15 (s, 1H, Ar-H4), 8.57 (1H, d, *J* = 4.5 Hz, pyrimidine-H), 8.71 (1H, s, triazole-H); ^13^C-NMR (DMSO-*d*_6_): δ 105.2, 112.7, 117.3, 119.4, 122.0, 130.2, 134.6, 136.8, 150.2, 152.5, 153.4, 155.8, 156.7, 164.0; MS *m*/*z* (%): 342, 344 (M^+^, M + 2, 83). Anal. Calcd for C_14_H_7_BrN_4_O_2_ (343.13): C, 49.00; H, 2.06; N, 16.33. Found: C, 48.88; H, 2.01; N, 16.24. 1H-, 13C-NMR Spectrum is in Supplementary Materials.

*6-Bromo-3-(tetrazolo[1,5-a]pyrimidin-7-yl)-2H-chromen-2-one (***7b**): Brown solid, 66% yield, mp 252–254 °C; IR (KBr) ν (cm^−1^): 3075 (=C–H), 2922 (–C–H), 1730 (C=O), 1606 (C=N); ^1^H-NMR (DMSO-*d*_6_) δ: 7.32 (d, 1H, *J* = 9.3 Hz, Ar-H8), 7.74 (d, 1H, *J* = 9.3 Hz, Ar-H7), 7.77 (s, 1H, Ar-H5), 8.06 (1H, d, *J* = 4.6 *Hz*, pyrimidine-H), 8.10 (s, 1H, Ar-H4), 8.53 (1H, d, *J* = 4.6 Hz, pyrimidine-H); MS *m*/*z* (%): 343, 345 (M^+^, M + 2, 82). Anal. Calcd for C_13_H_6_BrN_5_O_2_ (344.12): C, 45.37; H, 1.76; N, 20.35. Found: C, 45.32; H, 1.59; N, 20.28.

*6-Bromo-3-(2-phenylpyrazolo[1,5-a]pyrimidin-7-yl)-2H-chromen-2-one (***7c**): Brown solid, 68% yield, mp 203–205 °C; IR (KBr) ν (cm^−1^): 3140, 3068 (=C–H), 2919 (–C–H), 1734 (C=O), 1598 (C=N); ^1^H-NMR (DMSO-*d*_6_) δ: 7.34–7.56 (m, 6H, Ar-H), 7.72 (d, 1H, *J* = 9.2 Hz, Ar-H7), 7.91 (s, 1H, Ar-H5), 8.08 (1H, d, *J* = 4.5 Hz, pyrimidine-H), 8.18 (s, 1H, Ar-H4), 8.65 (1H, d, *J* = 4.5 Hz, pyrimidine-H), 8.86 (s, 1H, pyrazole-H); MS *m*/*z* (%): 417, 419 (M^+^, M + 2, 24). Anal. Calcd for C_21_H_12_BrN_3_O_2_ (418.24): C, 60.31; H, 2.89; N, 10.05. Found: C, 60.23; H, 2.75; N, 10.01.

*3-(Benzo[4,5]imidazo[1,2-a]pyrimidin-4-yl)-6-bromo-2H-chromen-2-one (***7d**): Yellow solid, 74% yield, mp 283–285 °C; IR (KBr) ν (cm^−1^): 3090, 3047 (=C–H), 2932 (–C–H), 1729 (C=O), 1607 (C=N); ^1^H-NMR (DMSO-*d*_6_) δ: 7.07–7.90 (m, 6H, Ar-H), 8.12 (1H, d, *J* = 4.5 Hz, pyrimidine-H), 8.34 (s, 1H, Ar-H5), 8.48 (1H, d, *J* = 4.5 Hz, pyrimidine-H), 8.63 (1H, (s, 1H, Ar-H4); MS *m*/*z* (%): 391, 393 (M^+^, M + 2, 100). Anal. Calcd for C_19_H_10_BrN_3_O_2_ (392.21): C, 58.18; H, 2.57; N, 10.71. Found: C, 58.12; H, 2.46; N, 10.53.

*6-Bromo-3-(8,10-dimethylpyrido[2',3':3,4]pyrazolo[1,5-a]pyrimidin-4-yl)-2H-chromen-2-one (***7e**): Brown solid, 69% yield, mp 252–254 °C; IR (KBr) ν (cm^−1^): 3092, 3056 (=C–H), 2930 (–C–H), 1731 (C=O), 1601 (C=N); ^1^H-NMR (DMSO-*d*_6_) δ: 2.60 (s, 3H, CH_3_), 2.89 (s, 3H, CH_3_), 7.08 (s, 1H, pyridine-H), 7.54 (d, 1H, *J* = 9.2 Hz, Ar-H8), 7.82 (d, 1H, *J* = 9.2 Hz, Ar-H7), 7.94 (1H, d, *J* = 4.5 Hz, pyrimidine-H), 8.17 (s, 1H, Ar-H5), 8.85 (1H, d, *J* = 4.5 *Hz*, pyrimidine-H), 8.91 (s, 1H, Ar-H4); ^13^C-NMR (DMSO-*d*_6_): δ 18.5, 20.5, 101.4, 110.2, 112.4, 116.8, 117.8, 118.5, 118.9, 119.4, 121.9, 128.5, 134.1, 135.2, 150.2, 150.9, 153.3, 155.3, 156.3, 165.2; MS *m*/*z* (%): 420, 422 (M^+^, M + 2, 96). Anal. Calcd for C_20_H_13_BrN_4_O_2_ (421.25): C, 57.02; H, 3.11; N, 13.30. Found: C, 57.14; H, 3.06; N, 13.24.

#### 3.2.4. Reactions of Enaminone **2** with Hydrazines

To a solution of the enaminone **2** (0.322 g, 1 mmol) in EtOH (10 mL) was added hydrazine hydrate (1 mL, 99%, 20 mmol) or phenylhydrazine (1 mL, 10 mmol) and the mixture was heated under reflux for 2 h. The reaction mixture was acidified with cold HCl and the formed product was filtered and crystallized from ethanol to give the respective pyrazoles **8a** and **8b**.

*6-Bromo-3-(1H-pyrazol-3-yl)-2H-chromen-2-one (***8a**): White solid, 78% yield, mp 217–219 °C; IR (KBr) ν (cm^−1^): 3286 (NH), 3127, 3091, 3051 (=C–H), 2928 (–C–H), 1730 (C=O), 1597 (C=N); ^1^H-NMR (DMSO-*d*_6_) δ: 6.74 (d, 1H, *J* = 7.6 Hz*,* pyrazole-H4), 7.34–7.43 (m, 2H, Ar-H), 7.77 (d, 1H, *J* = 7.5 Hz, pyrazole-H5), 7.96 (s, 1H, Ar-H5), 8.12 (s, 1H, Ar-H4), 11.42 (s, D_2_O-exchangeable, 1H, NH); ^13^C-NMR (DMSO-*d*_6_): *δ* 110.4, 118.3, 120.4, 123.5, 127.4, 129.1, 130.8, 134.7, 140.0, 142.5, 152.3 (Ar-C), 157.4 (C=O); MS *m*/*z* (%): 290, 292 (M^+^, M + 2, 100). Anal. Calcd for C_12_H_7_BrN_2_O_2_ (291.10): C, 49.51; H, 2.42; N, 9.62. Found: C, 49.48; H, 2.41; N, 9.47.

*6-Bromo-3-(1-phenyl-1H-pyrazol-3-yl)-2H-chromen-2-one (***8b**): white solid, 69% yield, mp 246–248 °C; IR (KBr) ν (cm^−1^): 3043, 3058 (=C–H), 2908 (–C–H), 1728 (C=O), 1597 (C=N); ^1^H-NMR (DMSO-*d*_6_) δ: 6.70 (d, 1H, *J* = 7.6 Hz*,* pyrazole-H4), 7.14–7.69 (m, 7H, Ar-H), 7.95 (d, 1H, *J* = 7.6 Hz*,* pyrazole-H5), 8.16 (s, 1H, Ar-H5), 8.36 (s, 1H, Ar-H4); MS *m*/*z* (%): 366, 368 (M^+^, M + 2, 100). Anal. Calcd for C_18_H_11_BrN_2_O_2_ (367.20): C, 58.88; H, 3.02; N, 7.63. Found: C, 58.64; H, 3.01; N, 7.48.

#### 3.2.5. Reactions of Enaminone 2 with Hydrazonoyl Chlorides **9a**, **9b** and Hydroximoyl Chlorides **10a**, **10b**

To a stirred solution of enaminone **2** (0.322 g, 1 mmol) and the appropriate hydrazonoyl chloride **9a** or **9b** or hydroximoyl chlorides **10a** or **10b** (1 mmol) in dry benzene (15 mL), an equivalent amount of triethylamine (0.15 mL 1 mmol) was added. The reaction mixture was heated under reflux for 4–6 h. The precipitated triethylamine hydrochloride was filtered off, and the filtrate was evaporated under reduced pressure. The residue was triturated with MeOH. The solid product, so formed in each case, was collected by filtration, washed with water, dried, and crystallized from EtOH to afford the corresponding pyrazole **12a**, **12b** and isoxazole derivatives **13a**, **13b**, respectively. The synthesized products together with their physical and spectral data are listed below.

*4-(6-Bromo-2-oxo-2H-chromene-3-carbonyl)-N,1-diphenyl-1H-pyrazole-3-carboxamide (***12a**): Brown solid, 67% yield, mp 223–225 °C; IR (KBr) ν (cm^−1^): 3412 (NH), 3111, 3073, 3018 (=C–H), 2903 (–C–H), 1728, 1682, 1640 (3 C=O), 1601 (C=N); ^1^H-NMR (DMSO-*d*_6_) δ: 7.13–7.73 (m, 12H, Ar-H), 8.17 (s, 1H, Ar-H5), 8.40 (s, 1H, pyrazole-H), 8.54 (s, 1H, Ar-H4), 12.05 (s, D_2_O-exchangeable, 1H, NH); ^13^C-NMR (DMSO-*d*_6_): *δ* 117.2, 117.6, 118.2, 123.8, 124.7, 129.3, 130.5, 131.1, 134.4, 135.9, 139.5, 140.2, 142.3, 145.7, 154.2, 156.1, 157.4, 175.6; MS *m*/*z* (%): 513, 515 (M^+^, M + 2, 60). Anal. Calcd for C_26_H_16_BrN_3_O_4_ (514.33): C, 60.72; H, 3.14; N, 8.17. Found: C, 60.59; H, 3.11; N, 8.04.

*6-Bromo-3-(1,3-diphenyl-1H-pyrazole-4-carbonyl)-2H-chromen-2-one (***12b**): Yellow solid, 69% yield, mp 192–194 °C; IR (KBr) ν (cm^−1^): 3111, 3073, 3018 (=C–H), 2902 (–C–H), 1727, 1640 (2 C=O), 1601 (C=N); ^1^H-NMR (DMSO-*d*_6_) δ: 7.18–7.69 (m, 12H, Ar-H), 8.15 (s, 1H, Ar-H5), 8.36 (s,1H, pyrazole-H), 8.47 (s, 1H, Ar-H4); MS *m*/*z* (%): 470, 472 (M^+^, M + 2, 83). Anal. Calcd for C_25_H_15_BrN_2_O_3_ (471.30): C, 63.71; H, 3.21; N, 5.94. Found: C, 63.64; H, 3.18; N, 5.79.

*3-(3-Benzoylisoxazole-4-carbonyl)-6-bromo-2H-chromen-2-one (***13a**): Yellow solid, 66% yield, mp 262–264 °C; IR (KBr) ν (cm^−1^): 3111, 3072, 3018 (=C–H), 2902 (–C–H), 1729, 1640 (2 C=O), 1601 (C=N); ^1^H-NMR (DMSO-*d_6_*) δ: 7.14–7.64 (m, 7H, Ar-H), 8.17 (s, 1H, Ar-H5), 8.38 (s, 1H, Ar-H4), 8.61 (s,1H, isoxazole-H); MS *m*/*z* (%): 423, 425 (M^+^, M+2, 100). Anal. Calcd for C_20_H_10_BrNO_5_ (424.20): C, 56.63; H, 2.38; N, 3.30. Found: C, 56.69; H, 2.24; N, 3.16.

*3-(3-(2-Naphthoyl)isoxazole-4-carbonyl)-6-bromo-2H-chromen-2-one (***13b**): Yellow solid, 74% yield, mp 186–188 °C; IR (KBr) ν (cm^−1^): 3110, 3073, 3020 (=C–H), 2915 (–C–H), 1728, 1673, 1639 (3 C=O), 1601 (C=N); ^1^H-NMR (DMSO-*d*_6_) δ: 7.27–8.18 (m, 10H, Ar-H), 8.42 (s, 1H, Ar-H4), 8.63 (s,1H, isoxazole-H); ^13^C-NMR (DMSO-*d*_6_): *δ* 116.5, 117.2, 118.4, 119.3, 119.8, 126.4, 127.8, 129.2, 129.7, 131.2, 132.5, 133.6, 134.2, 134.4, 134.7, 145.7, 153.2, 154.7, 158.6, 177.6, 187.4, 188.5; MS *m*/*z* (%): 473, 475 (M^+^, M + 2, 37). Anal. Calcd for C_24_H_12_BrNO_5_ (474.26): C, 60.78; H, 2.55; N, 2.95. Found: C, 60.49; H, 2.52; N, 2.76.

#### 3.2.6. Reaction of pyrazole **12a** with hydrazine hydrate

Hydrazine hydrate (80%, 2 mL) was added to a solution of the compound **12a** (1 mmol) in EtOH (10 mL). The reaction mixture was heated under reflux for 2 h, concentrated under vacuum, and diluted with water. The precipitate obtained was filtered off, washed with ice-cold water, dried and crystallized from EtOH to afford the pyrazolo[3,4-*d*]pyridazine **14** as yellow crystals in 70% yield; mp 280–282 °C; IR (KBr) ν (cm^−1^): 3346 (NH), 3110, 3054, 3020 (=C–H), 2921 (–C–H), 1728, 1653 (2C=O), 1606 (C=N); ^1^H-NMR (DMSO-*d*_6_) δ: 7.06–7.69 (m, 7H, Ar-H), 8.15 (s, 1H, Ar-H5), 8.38 (s, 1H, pyrazole-H), 8.59 (1H, (s, 1H, Ar-H4), 11.08 (s, 1H, D_2_O-exchangeable, NH); MS *m*/*z* (%): 434, 436 (M^+^, M + 2, 100). Anal. Calcd for C_20_H_11_BrN_4_O_3_ (435.23): C, 55.19; H, 2.55; N, 12.87. Found: C, 55.19; H, 2.55; N, 12.87.

#### 3.2.7. General Procedure for Synthesis of 1,3,4-Thiadiazole Derivatives **18a**–**h**

To a mixture of alkyl carbodithioate **3** (0.371 g, 1 mmol) and the appropriate hydrazonoyl halides **9a**–**h** (1 mmol) in ethanol (20 mL), triethylamine (0.5 mL) was added, the mixture was stirred at room temperature for 3 h. The resulting solid was collected and recrystallized from *N*,*N*-dimethylformamide to give the corresponding 1,3,4-thiadiazolines **18a**–**h**. The products **18a**–**h** together with their physical constants are listed below.

*5-((1-(6-Bromo-2-oxo-2H-chromen-3-yl)ethylidene)hydrazono)-N,4-diphenyl-4,5-dihydro-1,3,4-thiadiazole-2-carboxamide (***18a**)*.* Pale **y**ellow solid, 75% yield, mp 173–175 °C; IR (KBr) ν (cm^−1^): 3427 (NH), 3041 (=C–H), 2921 (–C–H), 1735, 1674 (2C=O), 1606 (C=N); ^1^H-NMR (DMSO-*d*_6_) δ: 3.27 (s, 3H, CH_3_), 7.35 (d, 1H, *J* = 9.2 Hz, Ar-H8), 7.40–7.80 (m, 10H, Ar-H), 7.85 (d, 1H, *J* = 9.2 Hz, Ar-H7), 8.19 (s, 1H, Ar-H5), 8.58 (s, 1H, Ar-H4), 11.84 (s, 1H, D_2_O-exchangeable, NH); MS, *m*/*z* (%) 559, 561 (M^+^, M + 2, 64). Anal. calcd for C_26_H_18_BrN_5_O_3_S (560.42): C, 55.72; H, 3.24; N, 12.50; found: C, 55.72; H, 3.24; N, 12.50.

*6-Bromo-3-(1-((3,5-diphenyl-1,3,4-thiadiazol-2(3H)-ylidene)hydrazono)ethyl)-2H-chromen-2-one (***18b**)*.* Yellow solid, 73% yield, mp 273–275 °C; IR (KBr) ν (cm^−1^): 3092, 3065, 3041 (=C–H), 2979, 2921 (–C–H), 1735, 1674 (2C=O), 1606 (C=N); ^1^H-NMR (DMSO-*d*_6_) δ: 3.27 (s, 3H, CH_3_), 7.08–7.83 (m, 12H, Ar-H), 8.19 (s, 1H, Ar-H5), 8.58 (s, 1H, Ar-H4); MS, *m*/*z* (%) 516, 518 (M^+^, M + 2, 52). Anal. calcd for C_25_H_17_BrN_4_O_2_S (517.40): C, 58.03; H, 3.31; 10.83; found: C, 58.01; H, 3.26; 10.67.

*Ethyl 5-((1-(6-bromo-2-oxo-2H-chromen-3-yl)ethylidene)hydrazono)-4-phenyl-4,5-dihydro-1,3,4-thiadiazole-2-carboxylate (***18c**). Yellow solid, 69% yield, mp 176–178 °C; IR (KBr) ν (cm^−1^): 3092, 3066, 3041 (=C–H), 2979, 2921 (–C–H), 1734, 1674 (C=O), 1505 (C=N); ^1^H-NMR (DMSO-*d*_6_) δ: 1.31 (t, 3H, *J* = 6.9 Hz, CH_2_CH_3_), 3.29 (s, 3H, CH_3_), 4.30 (q, 2H, *J* = 6.9Hz, CH_2_CH_3_), 7.40 (d, 1H, *J* = 9.1 Hz, Ar-H8), 7.43–7.67 (m, 5H, Ar-H), 7.84 (d, 1H, *J* = 9.1 Hz, Ar-H7), 8.18 (s, 1H, Ar-H5), 8.57 (s, 1H, Ar-H4); ^13^C-NMR (DMSO-*d*_6_): δ 13.5, 15.1, 61.8, 118.6, 119.4, 120.6, 122.6, 125.7, 127.2, 127.6, 128.4, 132.5, 135.4, 144.7, 146.3, 152.1, 153.7, 154.5, 160.2, 161.7; MS, *m*/*z* (%) 512, 514 (M^+^, M + 2, 73). Anal. calcd for C_22_H_18_BrN_4_O_4_S (513.36): C, 51.47; H, 3.34; N, 10.91; found: C, 51.47; H, 3.34; N, 10.91.

*Ethyl 5-((1-(6-bromo-2-oxo-2H-chromen-3-yl)ethylidene)hydrazono)-4-(p-tolyl)-4,5-dihydro-1,3,4-thiadiazole-2-carboxylate (***18d**). Yellow solid, 69% yield, mp 192–194 °C; IR (KBr) ν (cm^−1^): 3039 (=C–H), 2918 (–C–H), 1735, 1673 (2C=O), 1603 (C=N); ^1^H-NMR (DMSO-*d*_6_) δ: 1.29 (t, 3H, *J* = 6.9Hz, CH_2_CH_3_), 2.34 (s, 3H, CH_3_), 3.28 (s, 3H, CH_3_), 4.33 (q, 2H, *J* = 6.9Hz, CH_2_CH_3_), 7.41 (d, 1H, *J* = 9.1 Hz, Ar-H8), 7.46–7.66 (m, 4H, Ar-H), 7.83 (d, 1H, *J* = 9.1 Hz, Ar-H7), 8.18 (s, 1H, Ar-H5), 8.59 (s, 1H, Ar-H4); MS, *m*/*z* (%) 526, 528 (M^+^, M + 2, 20). Anal. calcd for C_23_H_19_BrN_4_O_4_S (527.39): C, 52.38; H, 3.63; N, 10.62; found: C, 52.26; H, 3.60; N, 10.51.

*Ethyl 5-((1-(6-bromo-2-oxo-2H-chromen-3-yl)ethylidene)hydrazono)-4-(4-chlorophenyl)-4,5-dihydro-1,3,4-thiadiazole-2-carboxylate (***18e**)*.* Yellow solid, 73% yield, mp 216–218 °C; IR (KBr) ν (cm^−1^): 3074 (=C–H), 2932 (–C–H), 1734, 1673 (2C=O), 1605 (C=N); ^1^H-NMR (DMSO-*d*_6_) δ: 1.33 (t, 3H, *J* = 6.9Hz, CH_2_CH_3_), 3.22 (s, 3H, CH_3_), 4.34 (q, 2H, *J* = 6.9Hz, CH_2_CH_3_), 7.41 (d, 1H, *J* = 9.1 Hz, Ar-H8), 7.43–7.66 (m, 4H, Ar-H), 7.85 (d, 1H, *J* = 9.1 Hz, Ar-H7), 8.20 (s, 1H, Ar-H5), 8.59 (s, 1H, Ar-H4); MS, *m*/*z* (%) 546, 548 (M^+^, M + 2, 20). Anal. calcd for C_22_H_16_BrClN_4_O_4_S (547.81): C, 48.23; H, 2.94; N, 10.23; found: C, 48.15; H, 2.84; N, 10.17.

*3-(1-((5-Acetyl-3-phenyl-1,3,4-thiadiazol-2(3H)-ylidene)hydrazono)ethyl)-6-bromo-2H-chromen-2-one (***18f**). Yellow solid, 73% yield, mp 228–230 °C; IR (KBr) ν (cm^−1^): 3092, 3066, 3041 (=C–H), 2979, 2921 (–C–H), 1734, 1674 (2C=O), 1505 (C=N); ^1^H-NMR (DMSO-*d*_6_) δ: 2.49 (s, 3H, CH_3_), 3.29 (s, 3H, CH_3_), 7.39 (d, 1H, *J* = 9.1 Hz, Ar-H8), 7.43–7.74 (m, 5H, Ar-H), 7.83 (d, 1H, *J* = 9.1 Hz, Ar-H7), 8.17 (s, 1H, Ar-H5), 8.56 (s, 1H, Ar-H4); MS, *m*/*z* (%) 482, 484 (M^+^, M+2, 63). Anal. calcd for C_21_H_15_BrN_4_O_3_S (483.34): C, 52.18; H, 3.13; N, 11.59; found: C, 52.12; H, 3.11; N, 11.46.

*3-(1-((5-Acetyl-3-(p-tolyl)-1,3,4-thiadiazol-2(3H)-ylidene)hydrazono)ethyl)-6-bromo-2H-chromen-2-one (***18g**). Yellow solid, 69% yield, mp 182–184 °C; IR (KBr) ν (cm^−1^): 3093, 3068, 3041 (=C–H), 2979, 2921 (–C–H), 1734, 1674 (2C=O), 1606 (C=N); ^1^H-NMR (DMSO-*d*_6_) δ: 2.28 (s, 3H, CH_3_), 2.49 (s, 3H, CH_3_), 3.26 (s, 3H, CH_3_), 7.39 (d, 1H, *J* = 9.1 Hz, Ar-H8), 7.42–7.68 (m, 4H, Ar-H), 7.84 (d, 1H, *J* = 9.1 Hz, Ar-H7), 8.19 (s, 1H, Ar-H5), 8.56 (s, 1H, Ar-H4); MS, *m*/*z* (%) 496 , 498 (M^+^, M + 2, 100). Anal. calcd for C_22_H_17_BrN_4_O_3_S (497.36): C, 53.13; H, 3.45; N, 11.26; found: C, 53.05; H, 3.42; N, 11.18.

*3-(1-((5-Acetyl-3-(4-chlorophenyl)-1,3,4-thiadiazol-2(3H)-ylidene)hydrazono)ethyl)-6-bromo-2H-chromen-2-one (***18h**). Yellow solid, 69% yield, mp 182–184 °C; IR (KBr) ν (cm^−1^): 3093, 3067, 3041 (=C–H), 2978, 2921 (–C–H), 1734, 1674 (2C=O), 1606 (C=N); ^1^H-NMR (DMSO-*d*_6_) δ: 2.49 (s, 3H, CH_3_), 3.26 (s, 3H, CH_3_), 7.41 (d, 1H, *J* = 9.1 Hz, Ar-H8), 7.47–7.68 (m, 4H, Ar-H), 7.85 (d, 1H, *J* = 9.1 Hz, Ar-H7), 8.20 (s, 1H, Ar-H5), 8.56 (s, 1H, Ar-H4); MS, *m*/*z* (%) 517, 519 (M^+^, M + 2, 100). Anal. calcd for C_21_H_14_BrClN_4_O_3_S (517.78): C, 48.71; H, 2.73; N, 10.82; found: C, 48.68; H, 2.59; N, 10.69.

#### 3.2.8. Alternative Synthesis of **18c**

To a solution of 3-acetyl-6-bromo-2*H*-chromen-2-one (**1**, 0.265 g, l mmol) in 2-propanol (10 mL), ethyl 5-hydrazono-4-phenyl-4,5-dihydro-1,3,4-thiadiazole-2-carboxylate (**19**, 0.264 g, 1 mmol) was added. The mixture was refluxed for 2 h then cooled to room temperature. The solid precipitated was filtered off, washed with water, dried and recrystallized from dimethylformamide to give the corresponding product, **18c** which were identical in all aspects (m.p., mixed m.p. and IR spectra) with those obtained from reaction of **3** with **9c** but in 69% yield.

#### 3.2.9. General Procedure for the Synthesis of 1,3-Thiazole Derivatives **23a**–**i**

A mixture of thiosemicarbazone **20** (0.338 g, 1 mmol) and the appropriate hydrazonoyl halides **9n**–**f** (1 mmol) in dioxane (20 mL) containing TEA (0.07 mL) was refluxed for 6 h, allowed to cool and the solid formed was filtered off, washed with EtOH, dried and recrystallized from DMF to give the corresponding thiazoles **23a**–**i**. The products **23a**–**i** together with their physical constants are listed below.

*6-Bromo-3-(1-(2-(4-methyl-5-(phenyldiazenyl)thiazol-2-yl)hydrazono)ethyl)-2H-chromen-2-one (***23a**). Red solid, 69% yield, mp 164–166 °C; IR (KBr) ν (cm^−1^): 3431 (NH), 3041 (=C–H), 2922 (–C–H), 1734 (C=O), 1600 (C=N); ^1^H-NMR (DMSO-*d*_6_) δ: 2.12 (s, 3H, CH_3_), 2.74 (s, 3H, CH_3_), 6.73–7.83 (m, 7H, Ar-H), 8.11 (s, 1H, Ar-H5), 8.33 (1H, (s, 1H, Ar-H4), 10.63 (s, 1H, D_2_O-exchangeable, NH); ^13^C-NMR (75 MHz, DMSO-*d*_6_): δ =10.3, 13.6 (CH_3_), 114.4, 116.2, 118.3, 119.9, 125.3, 127.4, 132.4, 136.5, 136.8, 138.7, 139.3, 142.2, 144.1, 144.5, 153.5, 157.6 (Ar-C), 163.4 (C=O); MS, *m*/*z* (%) 481, 483 (M^+^, M + 2, 46). Anal. calcd for C_21_H_16_BrN_5_O_2_S (482.35): C, 52.29; H, 3.34; N, 14.52. Found: C, 52.16; H, 3.21; N, 14.45%.

*6-Bromo-3-(1-(2-(4-methyl-5-(p-tolyldiazenyl)thiazol-2-yl)hydrazono)ethyl)-2H-chromen-2-one (***23b**). Red solid, 68% yield, mp 180–182 °C; IR (KBr) ν (cm^−1^): 3423 (NH), 3025 (=C–H), 2917 (–C–H), 1732 (C=O), 1599 (C=N); ^1^H-NMR (DMSO-*d*_6_) δ: 2.21 (s, 3H, CH_3_), 2.38 (s, 3H, CH_3_), 2.73 (s, 3H, CH_3_), 6.73–7.84 (m, 6H, Ar-H), 8.13 (s, 1H, Ar-H5), 8.33 (1H, (s, 1H, Ar-H4), 10.59 (s, 1H, D_2_O-exchangeable, NH); ^13^C-NMR (DMSO-*d*_6_): δ 11.6, 12.7, 20.5, 114.2, 118.3, 118.7, 120.6, 122.2, 127.4, 129.2, 129.7, 135.3, 137.1, 146.3, 149.5, 150.4, 151.8, 163.7, 164.8; MS, *m*/*z* (%) 495, 597 (M^+^, M + 2, 100). Anal. calcd for C_22_H_18_BrN_5_O_2_S (496.38): C, 53.23; H, 3.66; N, 14.11. Found: C, 53.09; H, 3.21; N, 14.03.

*6-Bromo-3-(1-(2-(5-((4-chlorophenyl)diazenyl)-4-methylthiazol-2-yl)hydrazono)ethyl)-2H-chromen-2-one (***23c**)*.* Red solid, 74% yield, mp 214–216 °C; IR (KBr) ν (cm^−1^): 3416 (NH), 3089 (=C–H), 2920 (–C–H), 1732 (C=O), 1595 (C=N); ^1^H-NMR (DMSO-*d*_6_) δ: 2.20 (s, 3H, CH_3_), 2.72 (s, 3H, CH_3_), 6.76–7.83 (m, 6H, Ar-H), 8.16 (s, 1H, Ar-H5), 8.34 (1H, (s, 1H, Ar-H4), 10.62 (s, 1H, D_2_O-exchangeable, NH); MS, *m*/*z* (%) 515, 517 (M^+^, M + 2, 100). Anal. calcd for C_21_H_15_BrClN_5_O_2_S (516.80): C, 48.81; H, 2.93; N, 13.55. Found: C, 48.79; H, 2.86; N, 13.50.

*6-Bromo-3-(1-(2-(4-methyl-5-(2-tolyldiazenyl)thiazol-2-yl)hydrazono)ethyl)-2H-chromen-2-one (***23d**)*.* Red solid, 69% yield, mp 189–191 °C; IR (KBr) ν (cm^−1^): 3411 (NH), 3064 (=C–H), 2920 (–C–H), 1732 (C=O), 1597 (C=N); ^1^H-NMR (DMSO-*d*_6_) δ: 2.20 (3H, s, CH_3_), 2.34 (s, 3H, CH_3_), 2.74 (s, 3H, CH_3_), 6.73–7.84 (m, 6H, Ar-H), 8.13 (s, 1H, Ar-H5), 8.33 (1H, (s, 1H, Ar-H4), 10.60 (s, 1H, D_2_O-exchangeable, NH); MS, *m*/*z* (%) 495, 497 (M^+^, M + 2, 70). Anal. calcd for C_22_H_18_BrN_5_O_2_S (496.38): C, 53.23; H, 3.66; N, 14.11. Found: C, 53.03; H, 3.27; N, 14.23.

*6-Bromo-3-(1-(2-(4-methyl-5-(m-tolyldiazenyl)thiazol-2-yl)hydrazono)ethyl)-2H-chromen-2-one (***23e**)*.* Red solid, 68% yield, mp 168–170 °C; IR (KBr) ν (cm^−1^): 3427 (NH), 3052 (=C–H), 2918 (–C–H), 1733 (C=O), 1598 (C=N); ^1^H-NMR (DMSO-*d*_6_) δ: 2.21 (s, 3H, CH_3_), 2.36 (s, 3H, CH_3_), 2.74 (s, 3H, CH_3_), 6.70–7.83 (m, 6H, Ar-H), 8.15 (s, 1H, Ar-H5), 8.34 (1H, (s, 1H, Ar-H4), 10.62 (s, 1H, D_2_O-exchangeable, NH); MS, *m*/*z* (%) 495, 597 (M^+^, M + 2, 34). Anal. calcd for C_22_H_18_BrN_5_O_2_S (496.38): C, 53.23; H, 3.66; N, 14.11. Found: C, 53.07; H, 3.17; N, 14.12.

*6-Bromo-3-(1-(2-(5-((4-methoxyphenyl)diazenyl)-4-methylthiazol-2-yl)hydrazono)ethyl)-2H-chromen-2-one (***23f**)*.* Red solid, 69% yield, mp 194–196 °C; IR (KBr) ν (cm^−1^): 3427 (NH), 3028 (=C–H), 2923 (–C–H), 1730 (C=O), 1596 (C=N); ^1^H-NMR (DMSO-*d*_6_) δ: 2.20 (s, 3H, CH_3_), 2.72 (s, 3H, CH_3_), 3.85 (s, 3H, OCH_3_), 6.76–7.79 (m, 6H, Ar-H), 8.13 (s, 1H, Ar-H5), 8.33 (1H, (s, 1H, Ar-H4), 10.63 (s, 1H, D_2_O-exchangeable, NH); MS, *m*/*z* (%) 511, 513 (M^+^, M + 2, 41). Anal. calcd for C_22_H_18_BrN_5_O_3_S (512.38): C, 51.57; H, 3.54; N, 13.67. Found: C, 51.52; H, 3.48; N, 13.49.

*6-Bromo-3-(1-(2-(5-((3-chlorophenyl)diazenyl)-4-methylthiazol-2-yl)hydrazono)ethyl)-2H-chromen-2-one (***23g**)*.* Red solid, 67% yield, mp 178–180 °C; IR (KBr) ν (cm^−1^): 3421 (NH), 3021 (=C–H), 2920 (–C–H), 1729 (C=O), 1593 (C=N); ^1^H-NMR (DMSO-*d*_6_) δ: 2.22 (s, 3H, CH_3_), 2.73 (s, 3H, CH_3_), 6.73–7.79 (m, 6H, Ar-H), 8.13 (s, 1H, Ar-H5), 8.34 (1H, (s, 1H, Ar-H4), 10.60 (s, 1H, D_2_O-exchangeable, NH); MS, *m*/*z* (%) 515, 517 (M^+^, M + 2, 35). Anal. calcd for C_21_H_15_BrClN_5_O_2_S (516.80): C, 48.81; H, 2.93; N, 13.55. Found: C, 48.81; H, 2.93; N, 13.55.

*6-Bromo-3-(1-(2-(5-((4-bromophenyl)diazenyl)-4-methylthiazol-2-yl)hydrazono)ethyl)-2H-chromen-2-one (***23h**). Orange solid, 74% yield, mp 193–195°C; IR (KBr) ν (cm^−1^): 3430 (NH), 3053 (=C–H), 2921 (–C–H), 1731 (C=O), 1600 (C=N); ^1^H-NMR (DMSO-*d*_6_) δ: 2.20 (s, 3H, CH_3_), 2.72 (s, 3H, CH_3_), 6.76–7.84 (m, 6H, Ar-H), 8.13 (s, 1H, Ar-H5), 8.34 (1H, (s, 1H, Ar-H4), 10.61 (s, 1H, D_2_O-exchangeable, NH); MS, *m*/*z* (%) 560, 562 (M^+^, M + 2, 84). Anal. calcd for C_21_H_15_Br_2_N_5_O_2_S (561.25): C, 44.94; H, 2.69; N, 12.48. Found: C, 44.83; H, 2.62; N, 12.30.

*6-Bromo-3-(1-(2-(4-methyl-5-((4-nitrophenyl)diazenyl)thiazol-2-yl)hydrazono)ethyl)-2H-chromen-2-one (***23i**)*.* Orange solid, 68% yield, mp 177–179 °C; IR (KBr) ν (cm^−1^): 3416 (NH), 3073 (=C–H), 2920 (–C–H), 1731 (C=O), 1596 (C=N); ^1^H-NMR (DMSO-*d*_6_) δ: 2.20 (s, 3H, CH_3_), 2.73 (s, 3H, CH_3_), 6.74–7.85 (m, 6H, Ar-H), 8.16 (s, 1H, Ar-H5), 8.34 (1H, (s, 1H, Ar-H4), 10.62 (s, 1H, D_2_O-exchangeable, NH); MS, *m*/*z* (%) 526, 528 (M^+^, M+, 73). Anal. calcd for C_21_H_15_BrN_6_O_4_S (527.35): C, 47.83; H, 2.87; N, 15.94. Found: C, 47.72; H, 2.80; N, 15.79.

### 3.3. Evaluation of the Antitumor Activity Using Viability Assay

Human hepatocellular carcinoma cell line (HEPG2) was obtained from the American Type Culture Collection (ATCC, Rockville, MD, USA). The cells were grown on RPMI-1640 medium supplemented with 10% inactivated fetal calf serum and 50 µg/mL gentamycin. The cells were maintained at 37 °C in a humidified atmosphere with 5% CO_2_ and were subcultured two to three times a week. Potential cytotoxicity of the compounds was evaluated on tumor cells using the method of Gangadevi and Muthumary [35]. The cells were grown as monolayers in growth RPMI-1640. The monolayers of 10^4^ cells adhered at the bottom of the wells in a 96-well microtiter plate incubated for 24 h at 37 °C in a humidified incubator with 5% CO_2_. The monolayers were then washed with sterile phosphate buffered saline (0.01 M pH 7.2) and simultaneously the cells were treated with 100 µL from different dilutions of tested sample in fresh maintenance medium and incubated at 37 °C. A control of untreated cells was made in the absence of tested sample. Positive controls containing doxorubicin drug was also tested as reference drug for comparison. Six wells were used for each concentration of the test sample. Every 24 h the observation under the inverted microscope was made. The number of the surviving cells was determined by staining the cells with crystal violet [36,37] followed by cell lysing using 33% glacial acetic acid and read the absorbance at 590 nm using microplate reader (SunRise, TECAN, Inc., San Diego, CA, USA) after well mixing. The absorbance values from untreated cells were considered as 100% proliferation. The number of viable cells was determined using microplate reader as previously mentioned before and the percentage of viability was calculated as [1 − (ODt/ODc)] × 100% where ODt is the mean optical density of wells treated with the tested sample and ODc is the mean optical density of untreated cells. The relation between surviving cells and drug concentration is plotted to get the survival curve of each tumor cell line after treatment with the specified compound. The 50% inhibitory concentration (IC_50_), the concentration required to cause toxic effects in 50% of intact cells, was estimated from graphic plots.

## 4. Conclusions

In the investigation described above, the 3-acetyl-6-bromo-2*H*-chromen-2-one moiety was introduced as a new class of antitumor agent against liver carcinoma. The scaffold has the advantage of facile synthetic protocol access. Briefly, target compounds were prepared via reactions of 6-bromo-3-(3-(dimethylamino)acryloyl)-2*H*-chromen-2-one, methyl 2-(1-(6-bromo-2-oxo-2*H*-chromen-3-yl)-ethylidene)hydrazine carbodithioate, 2-(1-(6-bromo-2-oxo-2*H*-chromen-3-yl)ethylidene) hydrazine-carbothioamide with *C*- and *N*-nucleophiles. The newly synthesized products were found to exhibit antitumor activities against liver carcinoma cell line (HEPG2-1) compared to doxorubicin as reference drug. Among all the test compounds, the most reactive compounds were the pyrazolo[1,5-*a*]pyrimidine **7c**, thiazole **23g** and 1,3,4-thiadiazole **18c** (IC_50_ = 2.70 ± 0.28, 3.50 ± 0.23 and 4.90 ± 0.69 µM, respectively).

## Supplementary Materials

Supplementary materials can be accessed at: http://www.mdpi.com /1420-3049/20/12/19803/s1.
