# Supplementary material for: Utility of 3-Acetyl-6-bromo-2H-chromen-2-one for the Synthesis of New Heterocycles as Potential Antiproliferative Agents
Source: Molecules. 2015 Dec 4;20(12):21826–39. doi: 10.3390/molecules201219803 (PMC6331961; doi:10.3390/molecules201219803)
Supplement: Supplementary file 1 [file molecules-20-19803-s001.pdf]

Supplementary Materials: Utility of 3-Acetyl-6-bromo-2*H*-chromen-2-one for Synthesis of New Heterocycles as Potential Antiproliferative Agents

Sobhi M. Gomha, Yasser H. Zaki and Abdou O. Abdelhamid

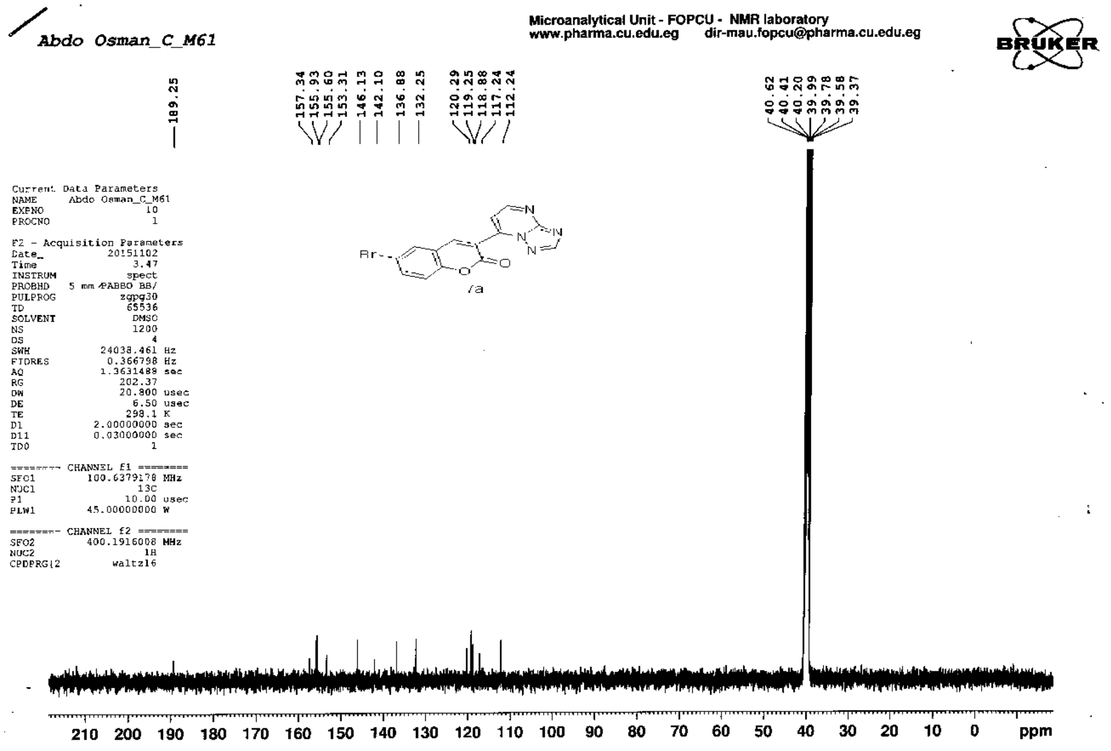

Figure S1. ^{13}C-NMR Spectrum of compound **7a**.

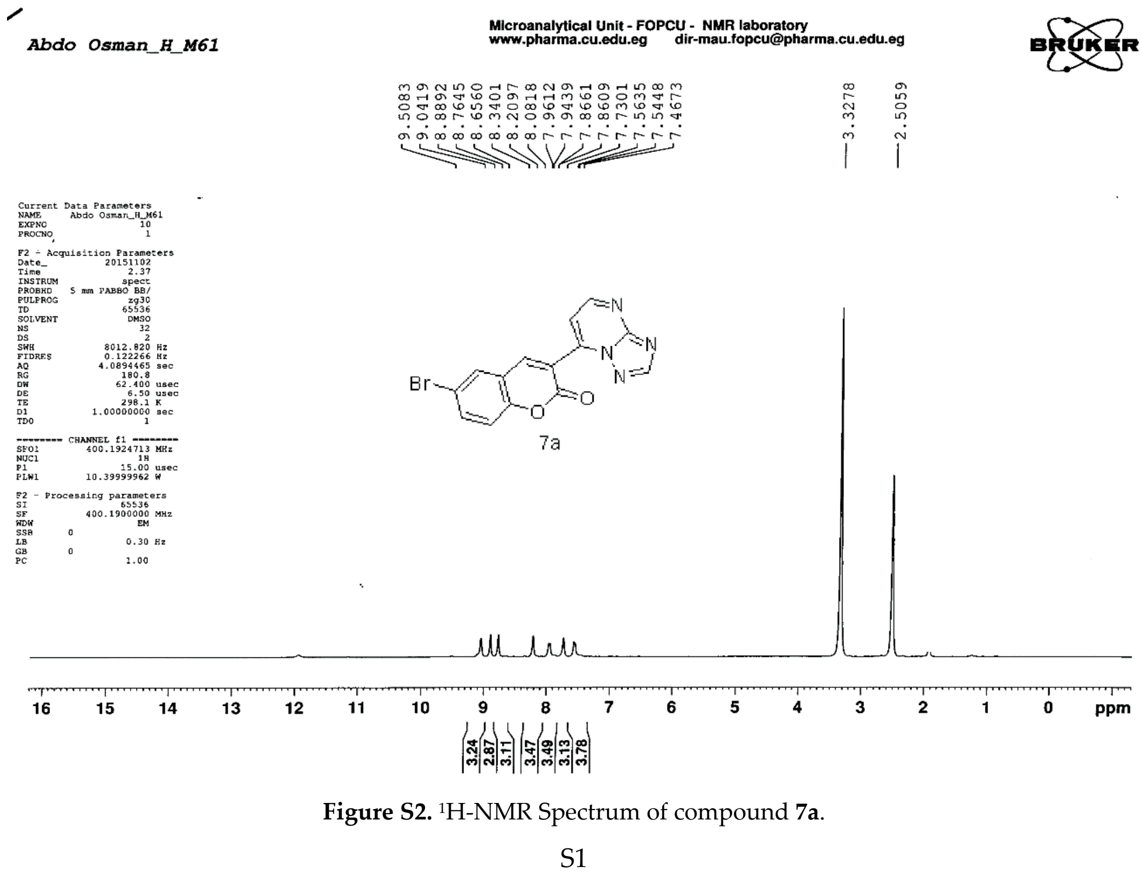

Figure S2. ^{1}H-NMR Spectrum of compound **7a**.

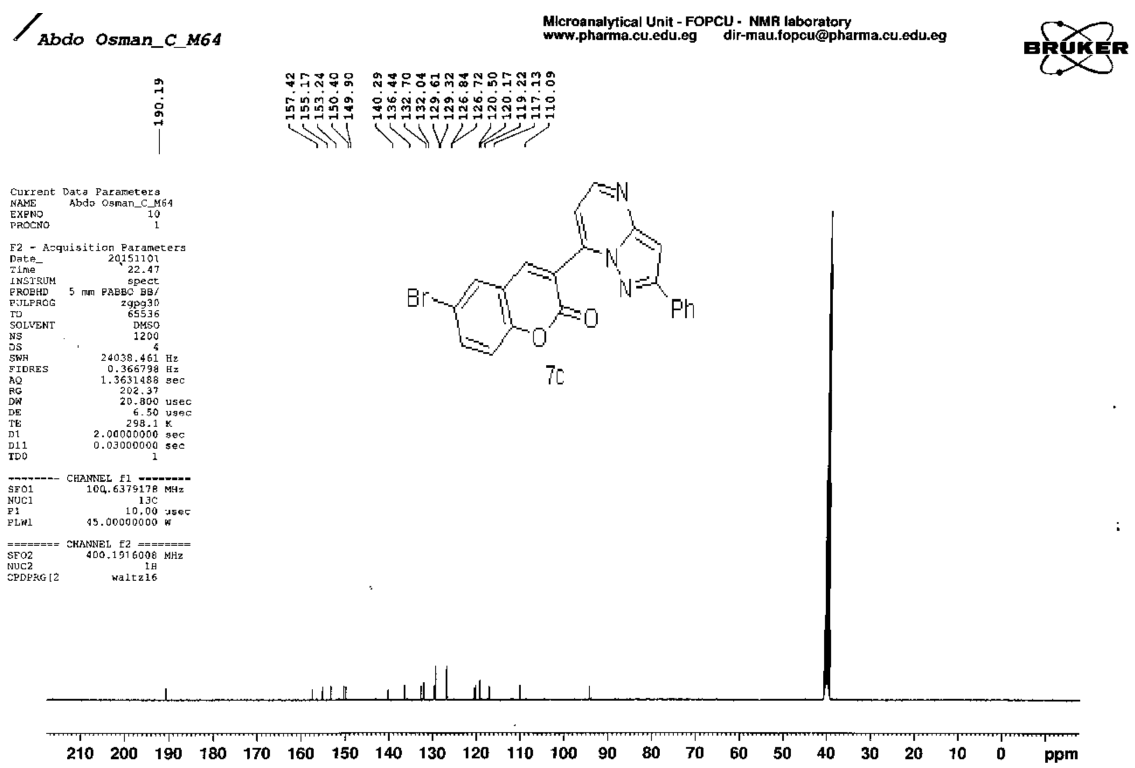

Figure S3. ^{13}C-NMR Spectrum of compound **7c**.

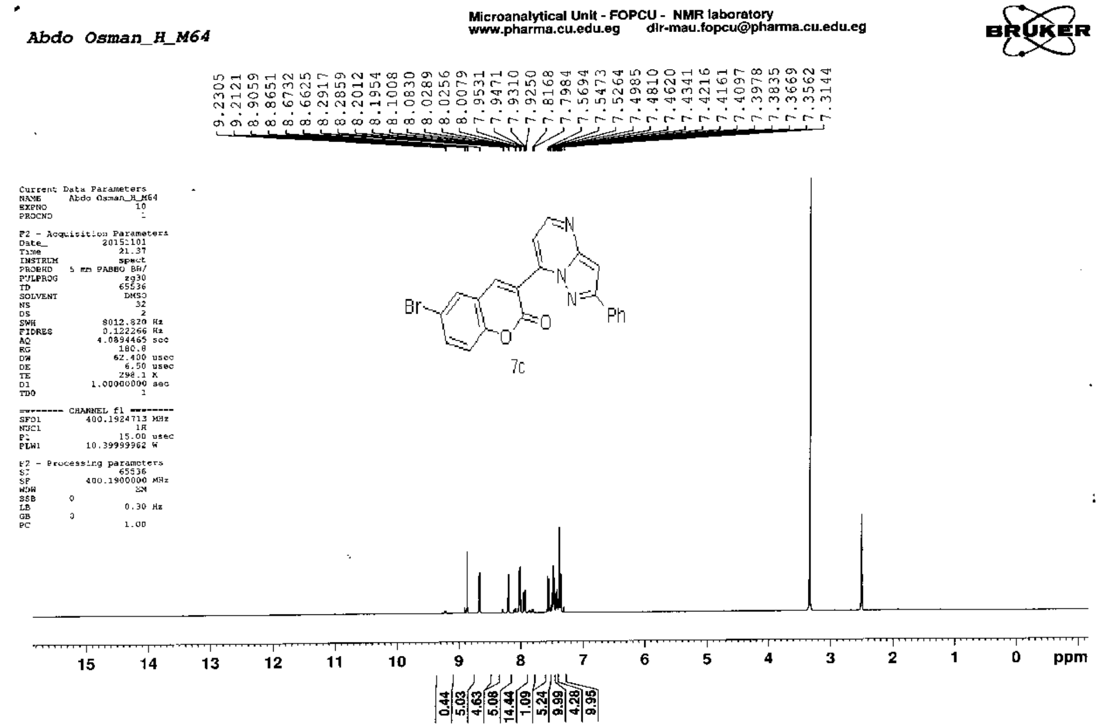

Figure S4. ^{1}H-NMR Spectrum of compound **7c**.

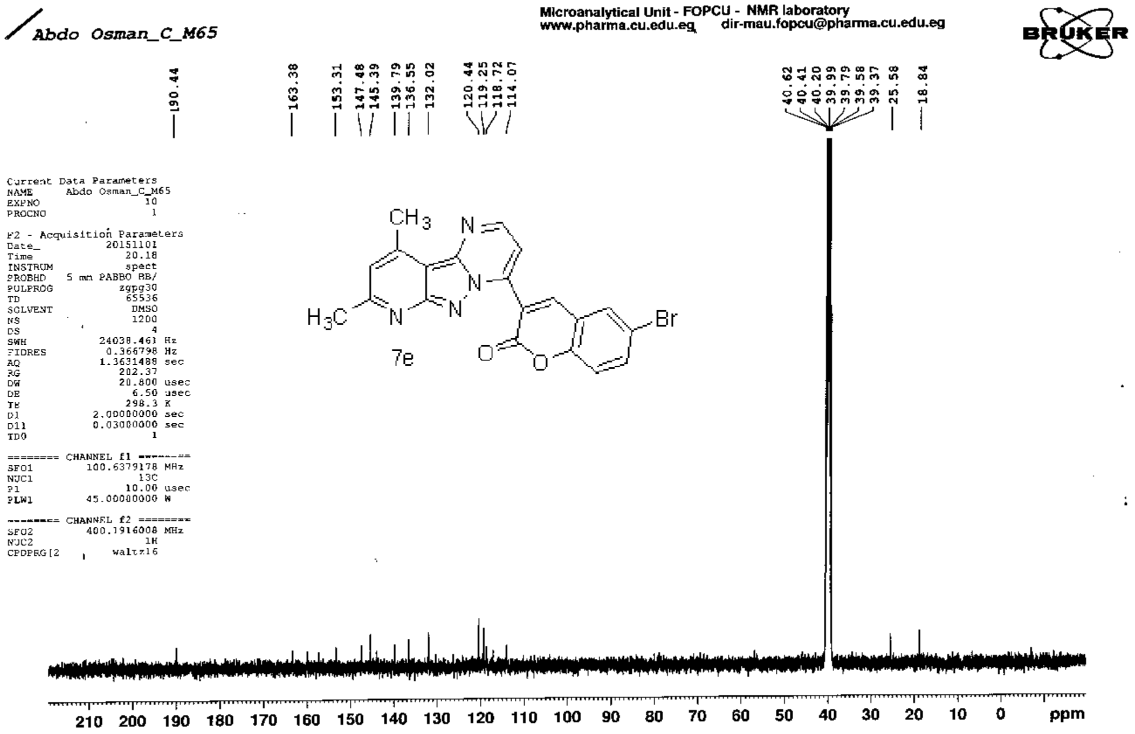

Figure S5. ^{13}C-NMR Spectrum of compound **7e**.

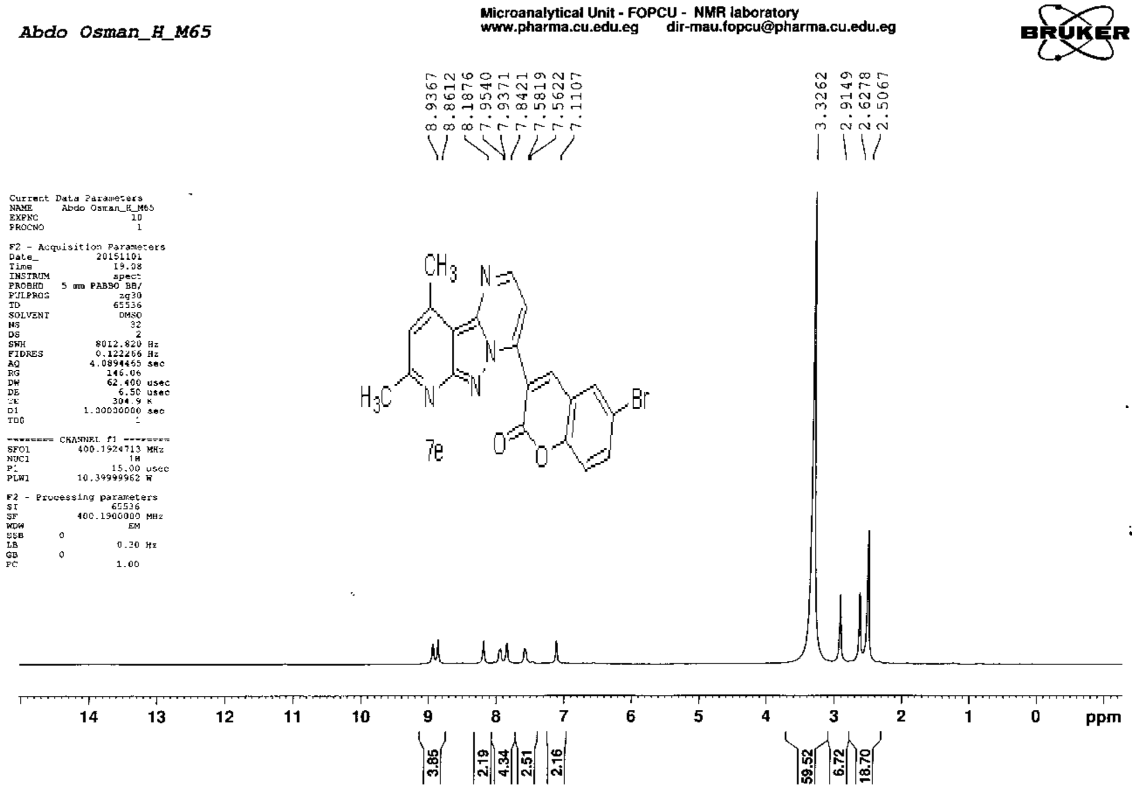

Figure S6. ^{1}H-NMR Spectrum of compound **7e**.

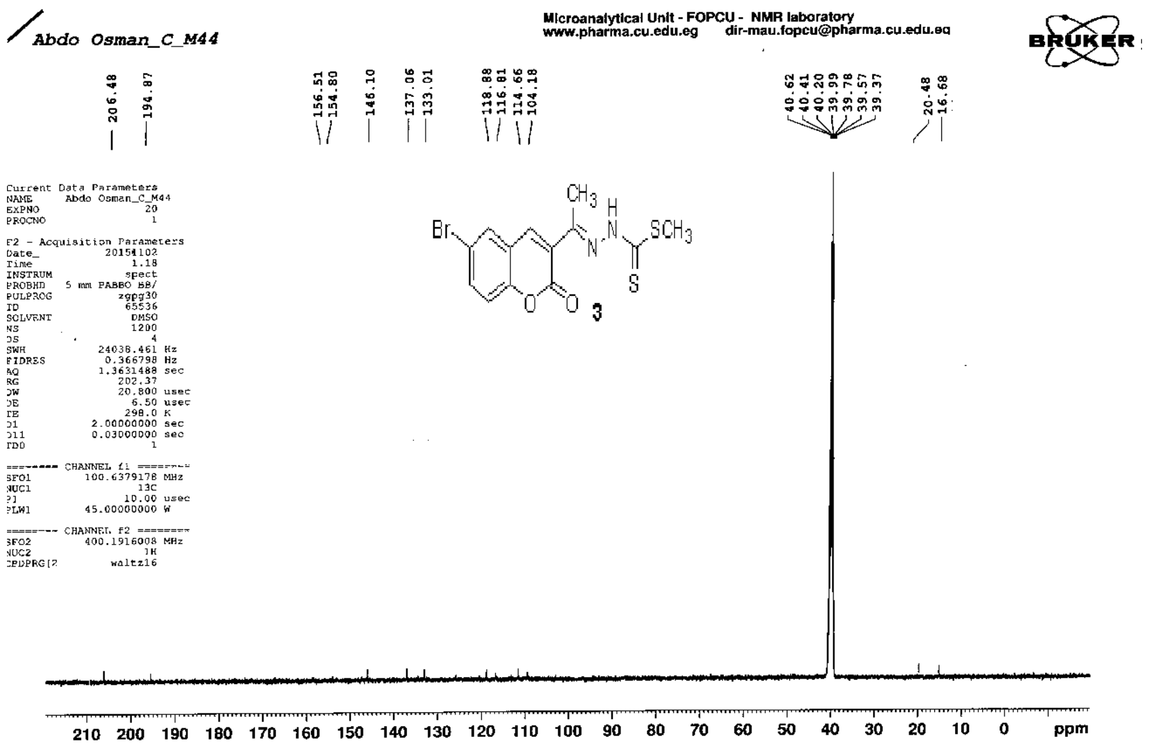

Figure S7. ^{13}C-NMR Spectrum of compound **3**.

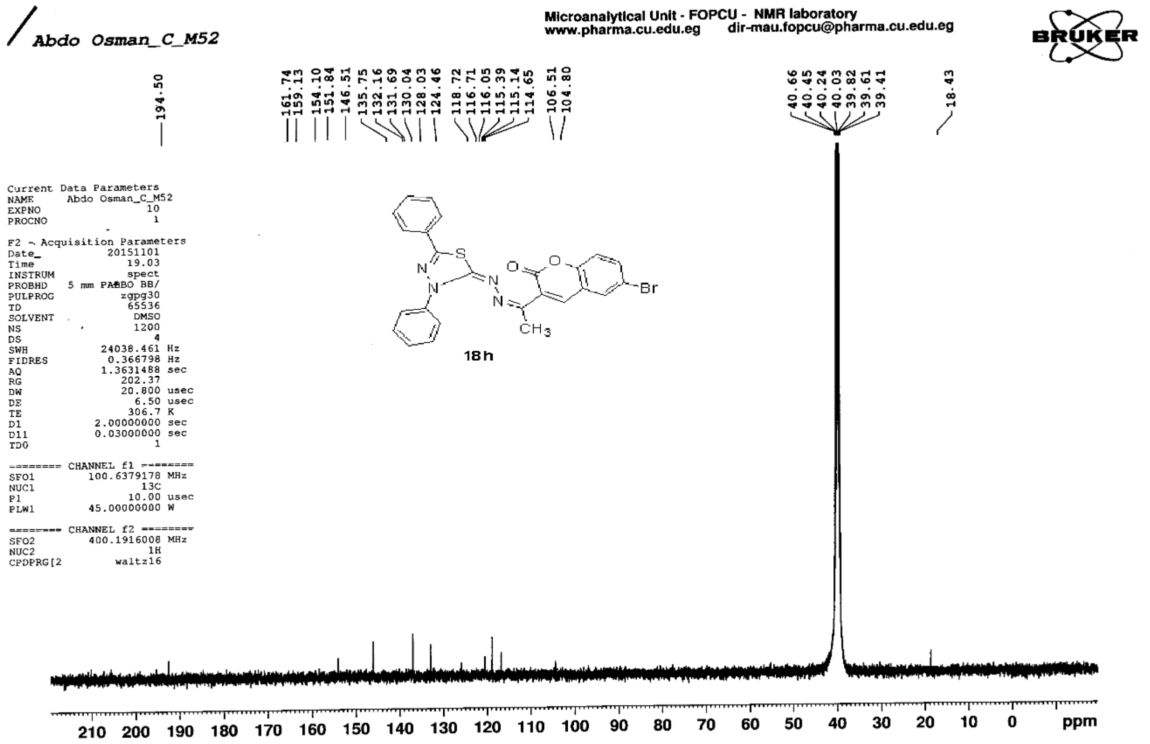

Figure S8. ^{13}C-NMR Spectrum of compound **18h**.

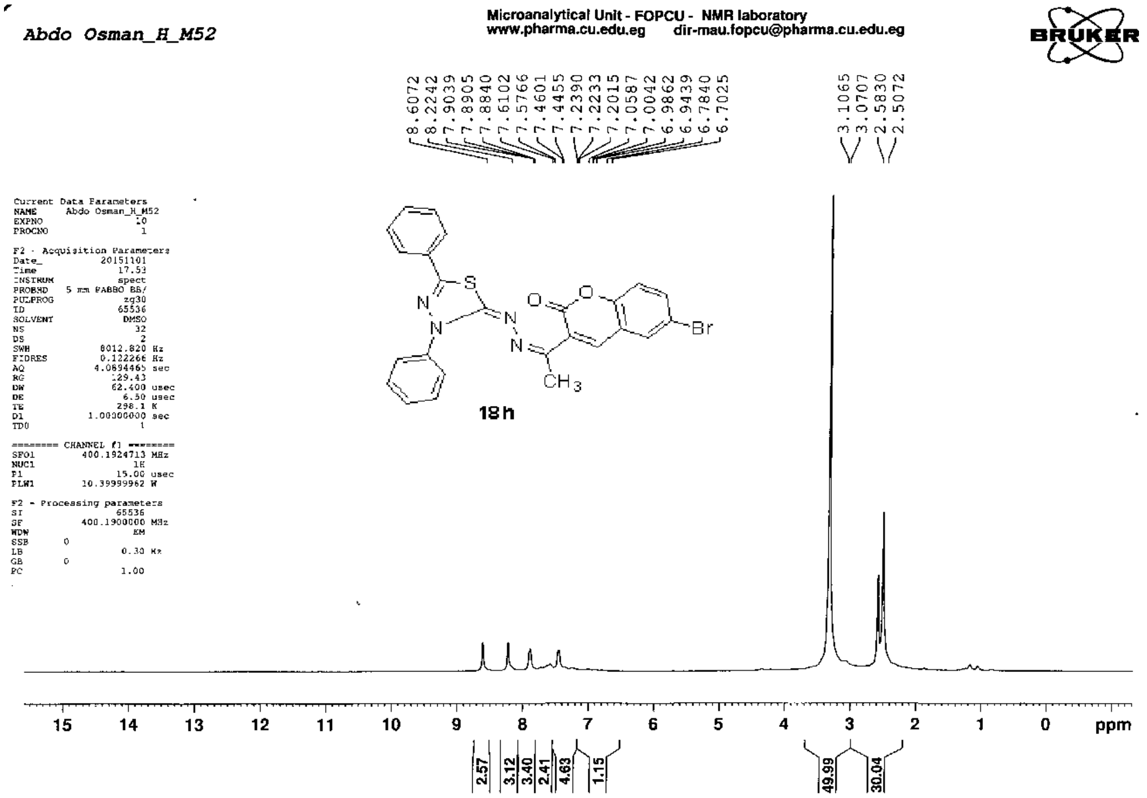

Figure S9. ^{1}H-NMR Spectrum of compound **18h**.

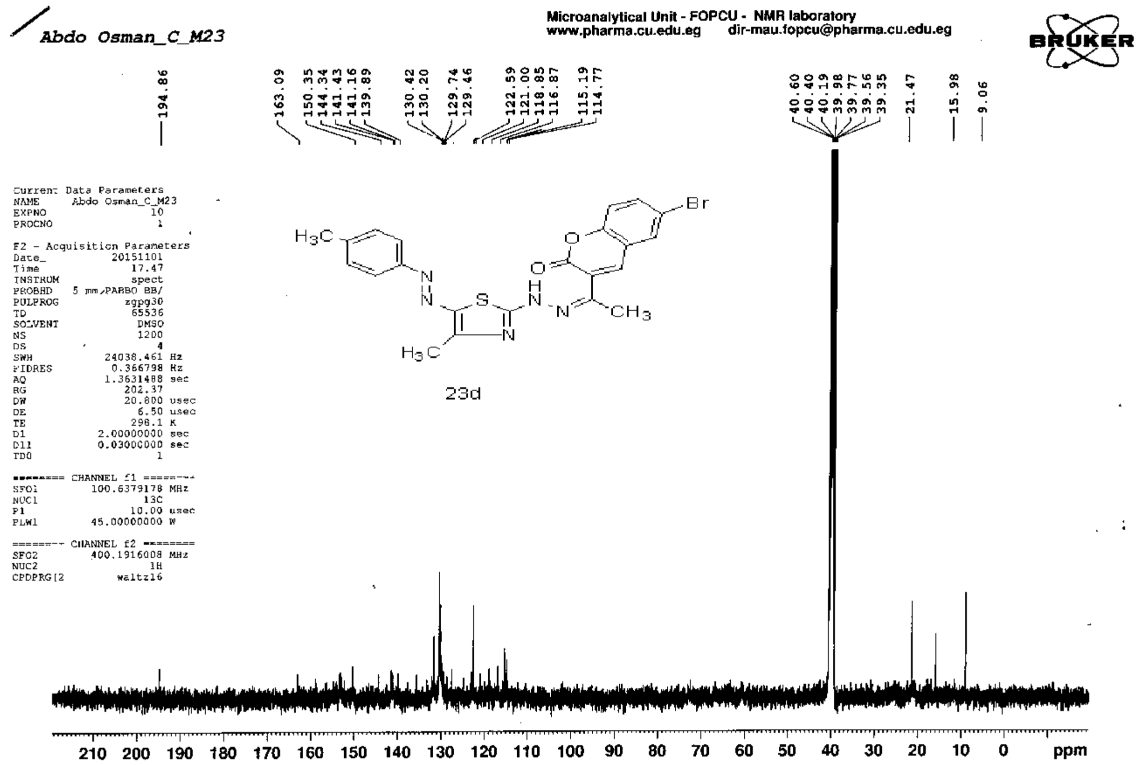

Figure S10. ^{13}C-NMR Spectrum of compound **23d**.

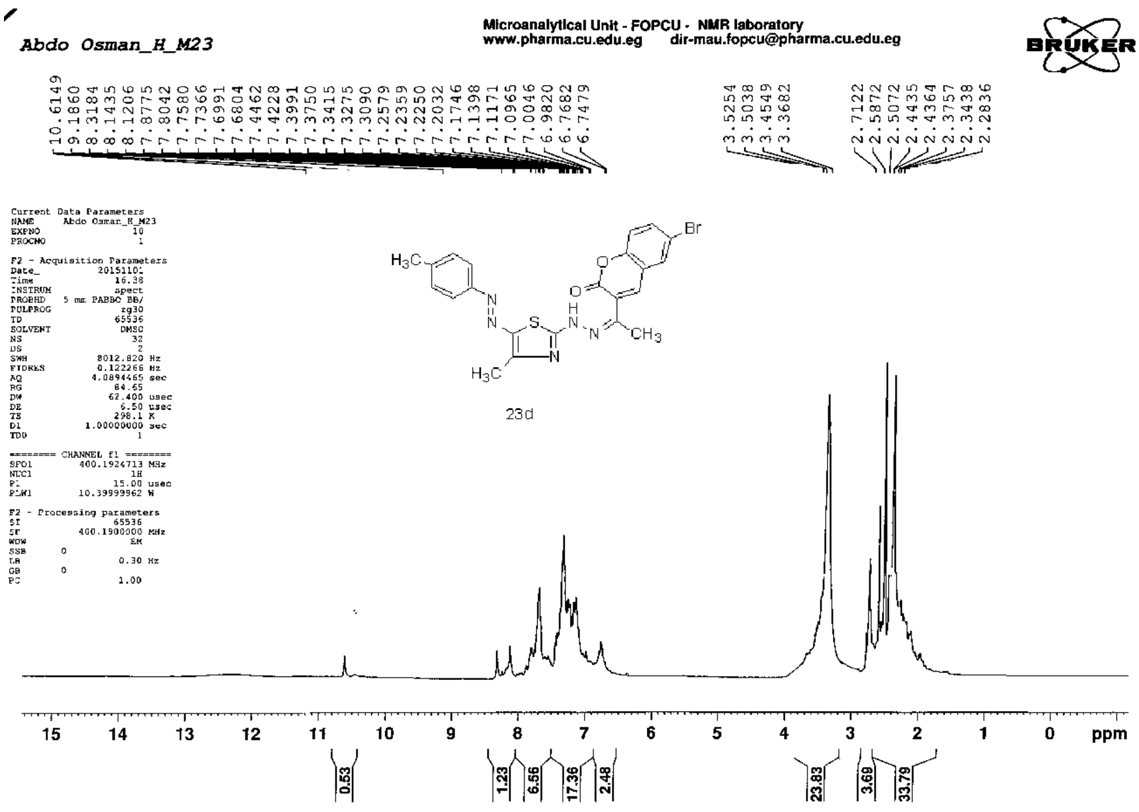

Figure S11. ^{1}H-NMR Spectrum of compound **23d**.

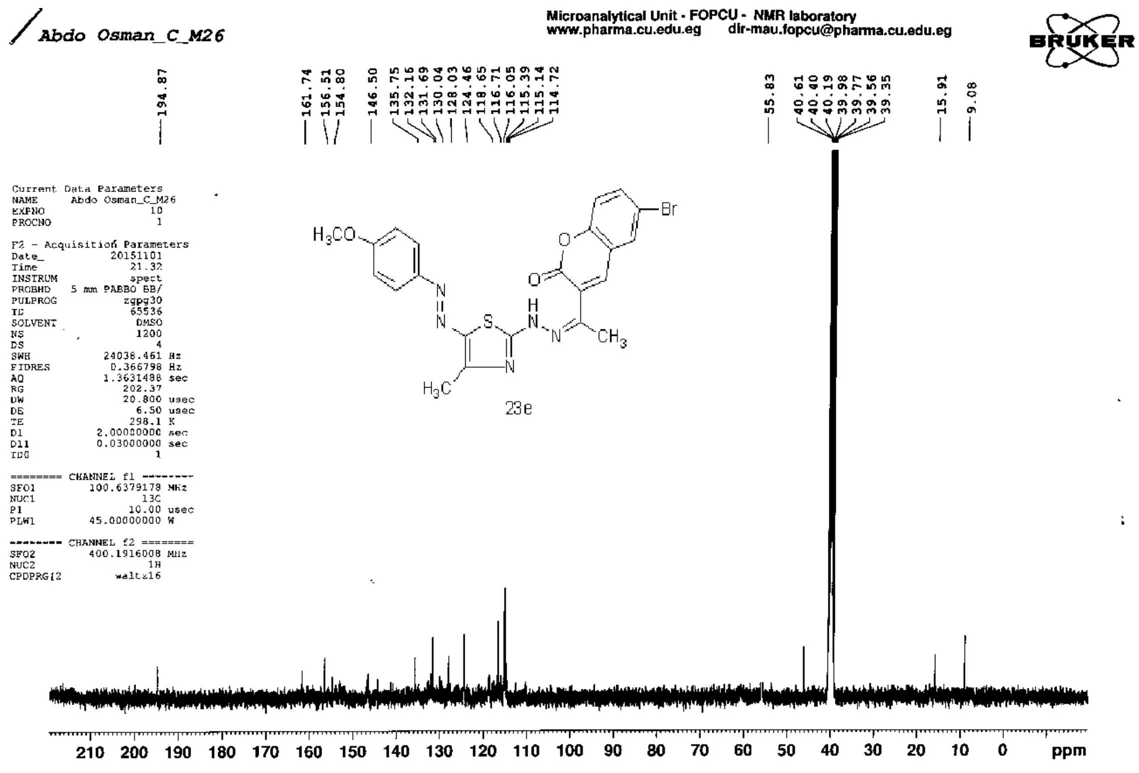

Figure S12. ^{13}C-NMR Spectrum of compound **23e**.

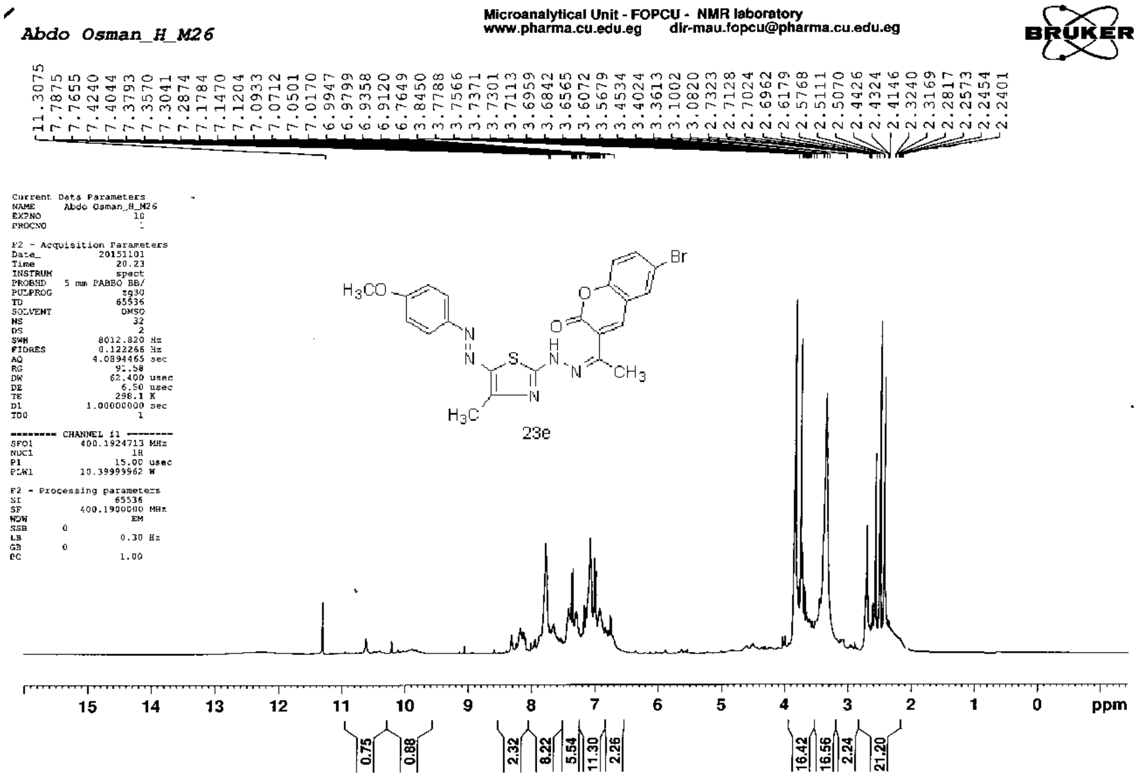

Figure S13. ^{1}H-NMR Spectrum of compound **23e**.

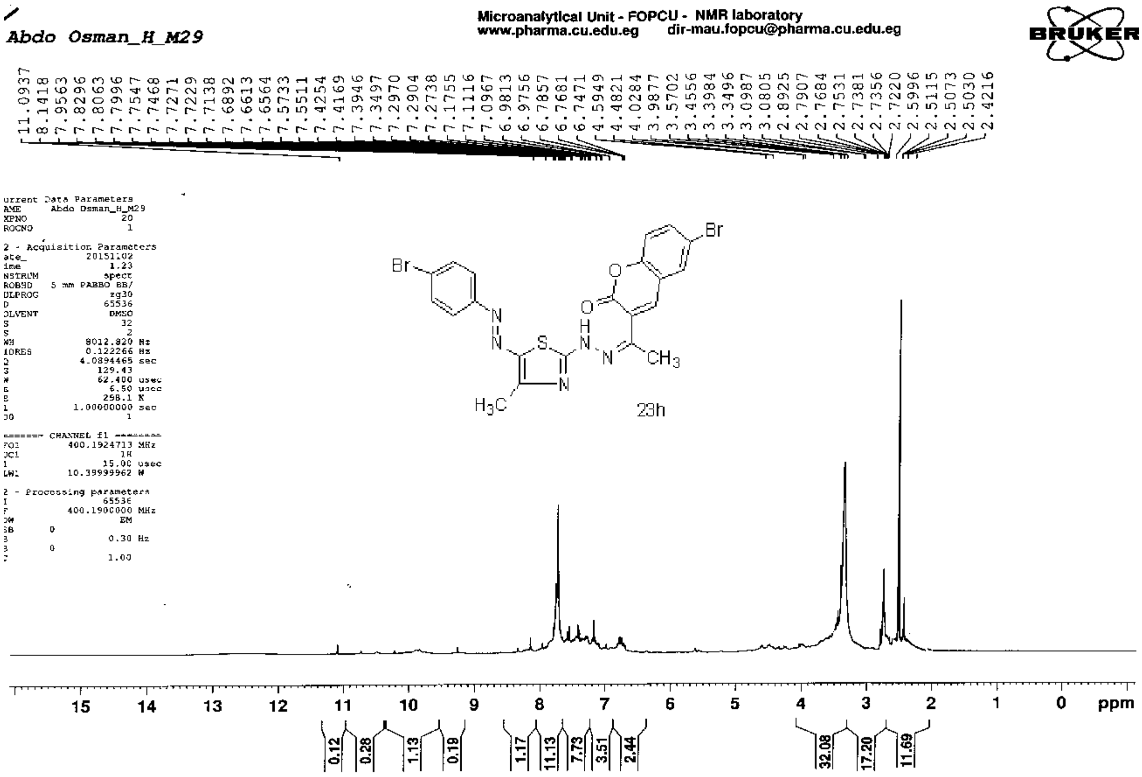

Figure S14. ^{1}H-NMR Spectrum of compound **23h**.
